# Initial WNT/β-Catenin or BMP Activation Modulates Inflammatory Response of Mesodermal Progenitors Derived from Human Induced Pluripotent Stem Cells

**DOI:** 10.3390/cells13211820

**Published:** 2024-11-04

**Authors:** Yulia Suzdaltseva, Anastasia Selezneva, Nikita Sergeev, Sergey L. Kiselev

**Affiliations:** Department of Epigenetics, Vavilov Institute of General Genetics of the Russian Academy of Sciences, 119333 Moscow, Russia

**Keywords:** human pluripotent stem cells, multipotent mesenchymal stromal cells, differentiation, inflammation, WNT/β-catenin, BMP, paraxial mesoderm, lateral mesoderm

## Abstract

Wound healing in adults largely depends on the functional state of multipotent mesenchymal stromal cells (MSCs). Human fetal tissues at the early stages of development are known to heal quickly with a full-quality restoration of the original structure. The differences in the molecular mechanisms that determine the functional activity of mesodermal cells in fetuses and adults remain virtually unknown. Using two independent human induced pluripotent stem cell (iPSC) lines, we examined the effects of the initial WNT and BMP activation on the differentiation of iPSCs via mesodermal progenitors into MSCs and highlighted the functions of these cells that are altered by the proinflammatory microenvironment. The WNT-induced mesoderm commitment of the iPSCs enhanced the expression of paraxial mesoderm (PM)-specific markers, while the BMP4-primed iPSCs exhibited increased levels of lateral mesoderm (LM)-specific genes. The inflammatory status and migration rate of the isogenic iPSC-derived mesoderm cells were assessed via gene expression analysis and scratch assay under the receptor-dependent activation of the proinflammatory IFN-γ or TNF-α signaling pathway. Reduced *IDO1* and *ICAM1* expression levels were detected in the WNT- and BMP-induced MSC progenitors compared to the isogenic MSCs in response to stimulation with IFN-γ and TNF-α. The WNT- and BMP-induced MSC progenitors exhibited a higher migration rate than isogenic MSCs upon IFN-γ exposure. The established isogenic cellular model will provide new opportunities to elucidate the mechanisms of regeneration and novel therapeutics for wound healing.

## 1. Introduction

The regenerative process is involved in the renewal, restoration, and growth of tissues after their loss during life or because of injury. Ideally, tissue regeneration is accomplished with the restoration of the complete original structure and functional activity. However, in an adult, complete restoration of damaged tissue does not occur, and healing often results in scar formation [1]. In some cases, disruption of the regeneration process leads to the development of chronic inflammation, keloids, and hypertrophic scars [2,3]. Although the cellular and molecular mechanisms underlying wound healing and scar formation are well described, the problems associated with pathological regenerative processes remain practically unresolved, and truly effective treatments still do not exist [4,5].

Mammalian fetal tissues throughout the first and second trimesters of development are known to heal quickly, with the restoration of the original tissue structure and typical cell differentiation and without scarring [6,7]. Obviously, the complete regeneration of fetal tissues occurs due to the coordinated action of mechanisms, the regulation of which is disturbed in healing and fibrosis. New approaches to the development of drugs aimed at stimulating the regenerative processes in tissues require the identification of the critical factors involved in the initiation and regulation of the intracellular signaling pathways that influence the inflammatory response and control the behavior of cells during fetal tissue repair. However, the physiological, cellular, and molecular mechanisms of fetal complete regeneration in humans remain poorly understood since such studies are strongly limited by ethical problems.

New technologies based on human pluripotent stem cells (hPSCs) provide a powerful platform, enabling the modeling of human development and genetic diseases [8,9]. The pluripotent state of embryonic stem cells (ESCs) and induced pluripotent stem cells (iPSCs) offers unprecedented opportunities for the modeling of sequential developmental events in the embryo in vitro. Real-time tracking of hPSC differentiation to obtain cell models corresponding to various stages of human development provides a level of experimental availability of embryonic stem cell-based models that is unattainable in vivo.

Tissue regeneration is impossible without the contribution of cells from fibroblastic histogenetic lineage of mesoderm, since they perform a regulatory function at the site of injury, stimulating cell recruitment, angiogenesis, synthesis, and the remodeling of the extracellular matrix necessary for the restoration of parenchyma. In particular, mesenchymal stromal cells (MSCs) have been shown to contribute to reductions in acute and chronic inflammation and the promotion of successful healing through the dynamically varying secretion of cytokines, chemokines, growth factors, extracellular vesicles, and extracellular matrix proteins under external signals from the microenvironment [10,11]. The complete regeneration of fetal tissues is logically assumed to occur with the involvement of the fetal progenitors of MSCs, which are phenotypically and functionally distinct from those of adults.

Although adult MSCs isolated from various tissues possess similar phenotypes, multilineage differentiation, and self-renewal potentials in vitro, they originate from developmentally diverse cell populations of lineage-specific mesodermal progenitors that arise from the neural crest, paraxial mesoderm, and lateral plate mesoderm [12]. The generation of mesodermal derivatives from iPSCs is initiated by mesoderm induction and the subsequent alternative mesodermal path navigation, which can be regulated by the signaling molecules ACTIVIN/NODAL, the wingless-related integration site (WNT), fibroblast growth factor (FGF), and bone morphogenetic protein (BMP) [13]. However, mesodermal patterning is dynamically controlled by the combinatorial effect of these signals that can act synergistically or antagonistically, depending on the context, resulting in different cellular responses. Moreover, signaling gradients can be formed by feedback loops from the same cells that differentiate in response to them, thus generating additional spontaneous heterogeneity in signaling activity [14,15]. The identification of the inductive and repressive signal cues that define the sequential steps, through which hPSCs elaborate the diversity of mesodermal progeny, facilitates the generation of mesodermal progenitors for MSCs [16]. However, mesodermal differentiation of hPSCs remains challenging because the current mesoderm induction protocols are obviously not yet perfect and fail to drive the entire iPSC population toward cells with the required mesodermal phenotype.

The functional heterogeneity of MSCs and their mesodermal progenitors can be determined not only by their ontogenetic origin but also by the inflammatory microenvironment. The immunomodulatory and fibrogenic activity of adult MSCs is mediated by the autocrine and paracrine effects of cytokines and growth factors, as well as the cell–cell and cell–matrix interactions through several positive and negative feedback loops that occur during the interaction of resident or migrated MSCs with immune cells at the site of acute and chronic inflammation [17,18]. Donor genetic backgrounds may also contribute to the functional heterogeneity of stem cells [19]. Several studies have confirmed the close similarity of the immunomodulatory activities between adult and hPSC-derived MSCs [20,21,22]. However, the functional activity of mesodermal progenitors derived from hPSCs in the inflammatory microenvironment has not yet been described. The generation of isogenic (genetically identical) cellular models corresponding to different stages of human development from one parental iPSC line will empower opportunities for the synchronous comparison of the phenotypic and functional features of adult and embryonic mesodermal cells to elucidate the mechanisms responsible for the complete healing of fetal tissues [23].

Here, we explored the effects of an initial activation of WNT/β-catenin and BMP signaling pathways on the in vitro differentiation of human iPSCs into paraxial and lateral mesoderm progenitors and their subsequent specification into MSCs. To gain more insights into the mechanisms underlying complete embryonic regeneration, we asked whether the ability of iPSC-derived MSC progenitors to respond to the proinflammatory signals differs from that of their isogenic MSCs. For this purpose, we attempted to unravel the effects of the proinflammatory microenvironment generated by interferon gamma (IFN-γ) or tumor necrosis factor alpha (TNF-α) on the functional activity of the iPSC-derived MSC progenitors. Gene expression analysis and a scratch assay were performed to examine the inflammatory status and migration rate of WNT- and BMP-induced MSC progenitors and their isogenic MSCs differentiated from iPSCs.

## 2. Materials and Methods

### 2.1. Human iPSC Culture

Two human iPSC lines, endo-iPSCs12 and RG2L, which were previously generated from HUVECs and the skin fibroblasts of healthy donors in our laboratory, were used in this study [24,25]. The iPSCs were cultured in a CO_2_ incubator at 37 °C in a humidified atmosphere with 5% CO_2_ in feeder-free culture conditions on standard plastic dishes for cell culture (Corning Costar, Corning, NY, USA), presorbed with Vitronectin XF solution (Stemcell Technologies, Toronto, ON, Canada) in ncTarget medium (Nuwacell Biotechnologies Co., Hefei, China). The medium was changed daily, and upon reaching 80% confluency, the iPSCs were passaged with ReLeSR reagent (Stemcell Technologies, Toronto, ON, Canada). During reseeding, the Rho kinase (ROCK) inhibitor Y-27632 (Stemcell Technologies, Toronto, ON, Canada) was added to the culture medium at a concentration of 5 μM for one day.

### 2.2. Mesodermal Differentiation of iPSCs

The iPSCs were split onto Vitronectin XF-coated dishes and cultured in ncTarget medium till 50% confluence. To induce mesoderm lineage differentiation, the pluripotency medium was substituted with the basal medium Dulbecco’s Modified Eagle Medium/Nutrient Mixture F-12 (DMEM/F12), containing 100 units/mL penicillin, 100 units/mL streptomycin, 2 mM glutamine, 100 µg/mL insulin, and 50 µg/mL transferrin (all PanEco, Moscow, Russia), followed by 10 ng/mL activin A (Thermo Fisher Scientific, Waltham, MA, USA) and 20 ng/mL bFGF (Merck Life Science LLC, Moscow, Russia); the cells were cultured overnight. Then, the iPSC lines were treated with 3 μM of the glycogen synthase kinase-3 inhibitor CHIR99021 (CHIR, Stemcell Technologies, Toronto, ON, Canada) at day 1 to mimic the initial WNT signaling of embryonic mesoderm development for the subsequent differentiation into paraxial mesoderm (PM). After 24 h, CHIR was removed to terminate the induced WNT signal, and the cells were subsequently incubated for 5 days in the same medium, which lacked bFGF but was supplemented with 15% KnockOut Serum Replacement (SR, Thermo Fisher Scientific, Waltham, MA, USA); the medium was replaced daily. In parallel to CHIR, 40 ng/mL BMP4 (Merck Life Science LLC, Moscow, Russia) was added to the predifferentiated iPSCs for downstream differentiation into lateral mesoderm (LM). Then, the cells were also incubated in the above medium for 5 days, but without removing BMP4. For further MSC development, the WNT- and BMP-induced progenitor cells were propagated in basal medium DMEM/F12 supplemented with 10% fetal bovine serum (FBS, Gibco, Thermo Fisher Scientific, Waltham, MA, USA).

### 2.3. Immunocytochemistry

The intercellular localization of β-catenin and HAND1 was assessed using immunofluorescence staining at day 0, 2, 6, and 21 of iPSC differentiation. Since CHIR was added to iPSCs once on day 1 of differentiation and removed from the medium after 24 h, β-catenin localization was assessed in cells de facto at days 0, 1, 5, and 20 after activation of WNT signaling. BMP was added to iPSCs synchronously with CHIR, but without removing from medium for 5 days. BMP4-treated iPSCs were stained for β-catenin in parallel with CHIR-primed cells. The cells were fixed in 4% paraformaldehyde (Thermo Fisher Scientific, Waltham, MA, USA) for 10–20 min at room temperature (RT) and then washed with phosphate buffer saline (PBS, PanEco, Moscow, Russia). After permeabilization with 0.5% Triton-X100 (Merck Life Science LLC, Moscow, Russia) for 10 min, the cells were blocked with 2.5% bovine serum albumin (BSA, Biosera, Shanghai, China) in PBS for 30 min. After overnight incubation at 4 °C with primary antibodies specific to β-catenin and HAND1, the cells were washed at least three times and incubated with ALEXA 488- or ALEXA 594-labeled secondary antibodies for 1 h at room temperature. The antibodies are summarized in Table S2. DAPI (Merck Life Science LLC, Moscow, Russia) was used for nuclear counterstaining. The images were obtained with fluorescence microscopy using the inverted microscope ZEISS Axio Observer with the Axiocam HRm and Axiovision 4.8 software (Carl Zeiss, Göttingen, Germany).

### 2.4. Differentiation of MSCs into Adipocytes, Osteoblasts, and Chondrocytes

The ability of MSCs to differentiate into adipogenic, osteogenic, and chondrogenic lineages was evaluated as described previously [26]. Briefly, to induce adipogenic differentiation, the MSCs were cultured in basal medium containing 10% equine serum (Biosera, Shanghai, China), 0.5 μM hydrocortisone, 0.5 mM isobutylmethylxanthine, and 60 μM indomethacin (all Merck Life Science LLC, Moscow, Russia) for 2–3 weeks. The medium was replaced weekly. The cells were stained with fresh Oil-Red O solution (Merck Life Science LLC, Moscow, Russia). The cell nuclei were counterstained with Mayer’s hematoxylin. Osteogenic differentiation of the MSCs was performed in serum-free DMEM/F12 supplemented with 0.2 mM ascorbate, 10 mM β-glycerophosphate, and 1 μM dexamethasone (Merck Life Science LLC, Moscow, Russia) for 2–3 weeks. To assess calcium accumulation and areas of mineralization, the cultures were stained with Alizarin Red (Merck Life Science LLC, Moscow, Russia). Chondrogenic differentiation of MSC was achieved in a three-dimensional aggregate culture exposed to DMEM/F12 supplemented with 10% FBS and 100 ng/mL transforming growth factor beta (TGF-β, Merck Life Science LLC, Moscow, Russia) for 2 weeks. Cryosections of the micromasses were stained with toluidine blue for the visualization of extracellular collagen. The samples were imaged using an Axio Scope microscope equipped with an Axiocam HRm camera and Axiovision 4.8 software (Carl Zeiss).

### 2.5. Reverse Transcription and Quantitative RT-PCR

Total RNA was extracted with TRIzol (Thermo Fisher Scientific, Waltham, MA, USA) from cells that had been cultured on Petri dishes. The Qubit 4 Fluorometer (Thermo Fisher Scientific, Waltham, MA, USA) was used to measure the concentration of the samples at the 260 nm absorbance wavelength using the Qubit™ RNA Broad Range (BR) Assay Kit (Thermo Fisher Scientific, Waltham, MA, USA). Electrophoresis of the 28S/18S bands was conducted to assess the quality of the total RNA samples. An MMLV RT kit (Evrogen, Moscow, Russia) was used to perform reverse transcription to cDNA. The RNA and DNA samples were stored at −20 °C. RT-PCR was conducted using the LightCycler 96 (Roche Diagnostics, Rotkreuz, Switzerland) amplification system and qPCRmix-HS-SYBR master mix (Evrogen, Moscow, Russia) containing SYBR Green I dye. The specific primers used are presented in Table S1. The visualization of nucleic acids in agarose gel (2%) was performed using the gel documentation imaging system E-BOX VX5/20M (Vilber, Marne-la-Vallée, France). The analysis of the differential expression of each gene was performed using the ΔΔCt method, and glyceraldehyde 3-phosphate dehydrogenase (*GAPDH*) served as an internal reference.

### 2.6. Flow Cytometry

Monolayer cell cultures were dissociated to single-cell suspensions with Versene solution (PanEco, Moscow, Russia) for 10 min at 37 °C in 5% CO_2_ and then collected via centrifugation at 300× *g* for 5 min. Then, all the steps were performed at 4 °C. For identification of the intracellular markers, the cells were fixed with 4% paraformaldehyde for 10 min. After washing with PBS, the cells were permeabilized with 90% methanol (Merck Life Science LLC, Moscow, Russia) for at least 30 min. Then, the cells were incubated in blocking solution (PBS supplemented with 2.5% BSA and 0.05% Tween 20 (Merck Life Science LLC, Moscow, Russia)) for 30 min. Staining with primary specific antihuman antibodies to HAND1 and DLL3 and sequential labeling with secondary antibodies conjugated with ALEXA 488 were performed for 1 h. The antibodies are summarized in Table S2. Every step was accompanied by washing with blocking solution at least three times to remove any unbound antibodies.

The exposure of surface proteins on the membrane was assessed via the immunocytochemical staining of cells using antihuman antibodies to CD73, CD90, CD105, CD14, CD34, and CD45, conjugated with the fluorochromes Alexa Fluor 647, allophycocyanin (APC), pyridine chlorophyll conjugated to cyanine 5.5 (PerCP_Cy5.5), and phycoerythrin conjugated to cyanine 7 (PE-Cy7), according to the manufacturer’s instructions. The corresponding isotype immunoglobulins were used as a negative control. The antibodies are summarized in Table S2. All the measurements were performed on a NovoCyte flow cytometer (ACEA Biosciences, Santa Clara, CA, USA) and LSRFortessa™ Cell Analyzer (BD Bioscience, San Jose, CA, USA) and analyzed using FlowJo v10 software.

### 2.7. Stimulation of Mesodermal Cells Differentiated from iPCSs with Inflammatory Factors

Mesodermal progenitor cells differentiated from the iPSCs treated with CHIR or BMP4 were generated according to above protocol on three 35 mm Petri dishes for each cell type for 6 days. The iPSC-derived MSCs were plated on three 35 mm Petri dishes and cultured in growth medium with the addition of 10% FBS until 90% confluence. At day 6, all the dishes were washed with DMEM/F-12 supplemented with 15% SR, and the cells were stimulated with individual inflammatory factors, namely human recombinant TNFα (20 ng/mL, Cloud-Clone corp., Beijing, China) or IFN-γ (20 ng/mL, Cloud-Clone corp., Beijing, China). After 48 h, the stimulated and nonstimulated cells were sampled for RNA isolation in lysis buffer and analyzed for gene expression using qRT-PCR, as described above.

### 2.8. Scratch Assays 

Isogenic mesodermal cells differentiated from iPSCs were grown under the same conditions as those described in the previous section. The cell layer was then scratched across the diameter of 35 mm Petri dishes using 200 μL pipette tips (Greiner Bio-One, Kremsmunster, Austria). The culture medium was then immediately removed (along with any dislodged cells) and replaced with 1.5 mL of DMEM/F-12 containing 15% SR, with the addition of 20 ng/mL of TNFα or IFN-γ. Images from the same viewpoint were obtained under microscopy at the baseline (0 h) and every 24 h after wounding for determination of the extent of wound closure. ImageJ 1.49v (National Institutes of Health, Bethesda, MD, USA) was used to measure the void area, and the extent of the wound closure was evaluated (width (%) = void area /baseline area) in each group.

### 2.9. Statistical Analysis

Statistical analyses were performed using SPSS Statistics 25.0 software (SPSS Inc., Chicago, IL, USA). Statistical comparisons of two datasets at the same time point were performed using a paired *t*-test, and for the time courses, differences within one group over time were assessed using one-way ANOVA. The chi-squared (χ2) test was used for qualitative data analysis of the cell populations in independent groups. The Pearson correlation coefficient was used to evaluate sample similarity. The data were obtained from at least three independent experiments. The measurement data were represented as the mean and standard deviation. Statistical differences were considered significant at *p* < 0.05.

## 3. Results

### 3.1. Mesodermal Specification of iPSCs Through Activation of WNT and BMP Signaling Pathways

We established our protocol for the mesodermal specification of iPSCs based on the previous studies, which reported the stepwise methods for the efficient differentiation of hPSCs into paraxial mesoderm (PM) and lateral mesoderm (LM) through the activation of WNT and/or BMP signaling [27,28,29,30,31,32,33,34]. We also considered the data of Loh KM et al., who demonstrated that WNT and BMP signals may drive the bifurcation of the primitive streak to the PM versus LM, respectively [16].

In the present study, two human iPSC lines, endo-iPS12 and RG2L, which had been previously established in our laboratory, were used for the generation of isogenic cell models corresponding to different stages of human development, with sequential differentiation of the iPSCs through the PM and LM of the human embryo into MSCs.

**Figure 1:**
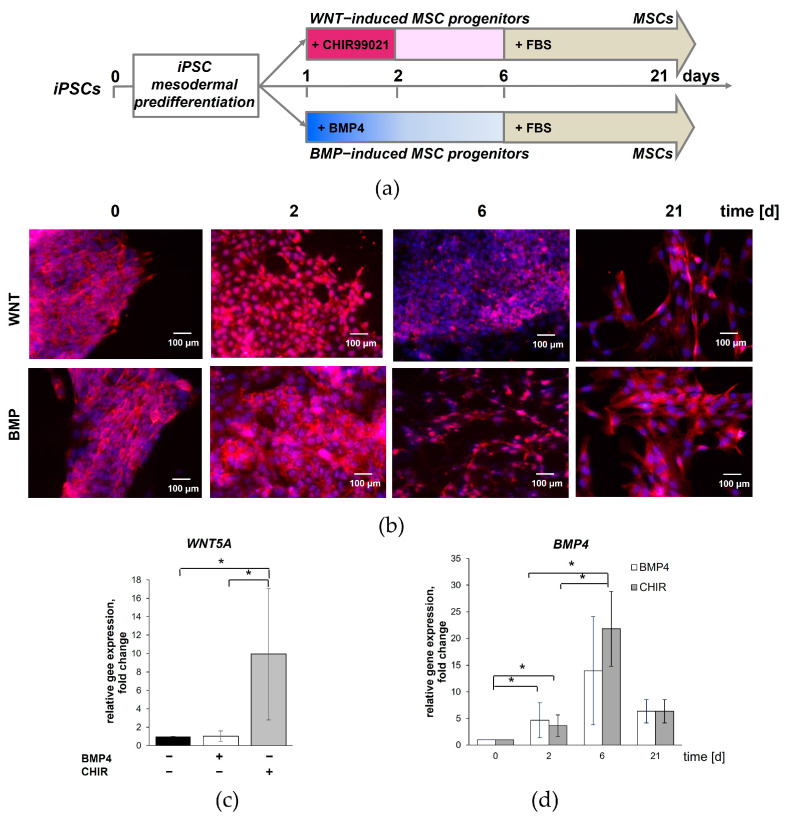
Activation of WNT and BMP signaling pathways during mesoderm specification of iPSCs. (**a**) Schematic representation of the iPSC differentiation protocol. Subconfluent iPSC cultures were induced into mesoderm differentiation by deprivation of pluripotency medium. Switching to PM and LM specifications was induced by CHIR or BMP4 treatment, respectively. MSCs were developed from the WNT- and BMP-induced progenitors by propagation in a serum-containing medium. (**b**) Immunostaining shows intercellular localization of β-catenin (red) in cells during mesodermal differentiation of the iPSCs. Nuclei are stained with DAPI. Scale bar, 100 μm. (**c**) Relative mRNA expression for *WNT5A* shows increased transcript levels in the cells treated with CHIR compared with counterparts treated with BMP4 or untreated iPSCs on day 2 of mesodermal differentiation. (**d**) Cells treated with CHIR and BMP4 have similar relative expression to *BMP4*, with the maximum on day 6 of mesodermal differentiation. Data are represented as mean ± SD, *n* = 3 independent experiments. *t*-test, * reliability of differences at *p* < 0.05.

We previously showed that deprivation of the pluripotency medium and supplemental treatment with bFGF and activin A led to mesodermal specification of iPSCs, which resulted in the appearance of a cell population coexpressing the primitive streak markers *TBXT* and *MIXL1* [27]. Thus, we initiated mesodermal predifferentiation of the iPSCs to a primitive streak at day 0. After 24 h, the bifurcation of the primitive streak cells was induced by BMP4 for the LM specification and by CHIR for the PM. Then, the MSCs were developed from the WNT- and BMP-induced progenitors to produce isogenic cell models corresponding to the different stages of human development derived from the iPSCs. The experimental design is shown in Figure 1a.

As WNT/β-catenin and BMP signaling pathways are involved in embryonic mesoderm development through the induction of differentiation and alternative pathway navigation, we first assessed the canonical β-catenin-mediated WNT pathway activation by imaging the localization of endogenous β-catenin in the cells differentiated from the iPSCs. Initially, the iPSCs expressed a high level of membrane-associated β-catenin. The activation of WNT signaling by CHIR in the cells on the second day of the experimental protocol was induced in almost all of the cells (Figure 1b). At the same time, the nuclear translocation of β-catenin was observed only in single cells when BMP4 was added to the cell culture. Subsequently, the nuclear localization of β-catenin declined in both types of cells over the next few days. By day 6, the progenitors derived from the BMP4-treated iPSCs had similar fibroblast-like morphology and intercellular localization of β-catenin compared to their isogenic MSCs; however, they were significantly smaller in size. At the same time, the CHIR-treated cells were still similar in the localization of β-catenin to the parenteral iPSCs; however, they were larger in size (Figure 1b).

In addition to the canonical β-catenin-mediated WNT pathway, the noncanonical β-catenin-independent WNT pathway can also regulate early developmental events toward mesodermal cell fate. The canonical WNT pathway mainly controls cell proliferation, whereas the noncanonical WNT pathways regulate cell polarity and migration, and the two main pathways form a network of mutual regulation. Noncanonical Wnt5a regulates evolutionarily conserved planar cell polarity signaling and anterior-posterior axis elongation in mammalian development. Anterior-posterior axis elongation requires continuous generation and proper organization of paraxial mesoderm [35,36]. We aimed to determine whether WNT5a expression is activated in cells during mesodermal differentiation of iPSCs. qRT-PCR revealed that the mRNA level of *WNT5A* was markedly upregulated in the CHIR-treated cells on day 2 when compared to the undifferentiated iPSCs and the cells treated with BMP4 (Figure 1c). The initiation of iPSC commitment with either CHIR or BMP4 significantly increased *BMP4* expression in the cells during mesodermal differentiation, with the maximum being reached on day 6 (Figure 1d). qRT-PCR analysis did not reveal significant changes in the level of β-catenin expression during the mesodermal differentiation of the iPSCs.

Overall, the mRNA and protein analysis demonstrated that the early exposure to CHIR or BMP4 enhances and navigates the mesodermal differentiation of iPSCs.

### 3.2. Derivation of MSCs from Human Pluripotent Stem Cell Through Mesodermal Specification by CHIR and BMP4

**Figure 2:**
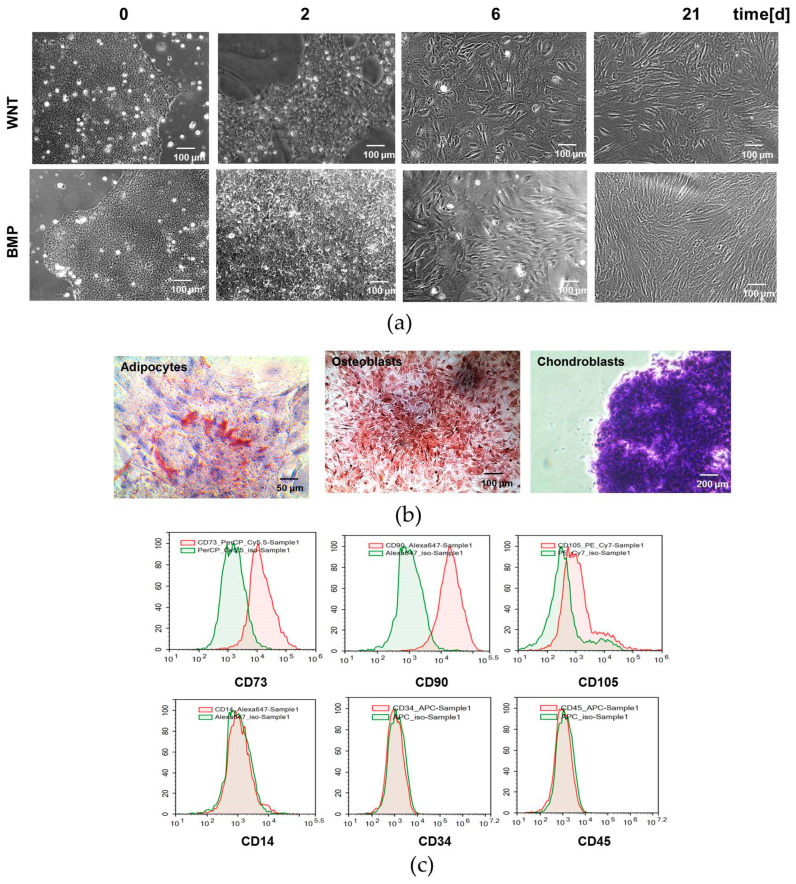
Differentiation of iPSCs into MSCs. (**a**) Brightfield microscopy images of cells during mesodermal differentiation of the iPSCs. (**b**) Differentiation of the iPSC-derived MSCs into adipocytes, osteoblasts, and chondroblasts. Accumulation of fat vacuoles (stained with oil red and hematoxylin), accumulation of mineralized matrix (stained with alizarin red and hematoxylin), formation of cartilaginous micromass (cryosections stained with toluidine blue). (**c**) Flow cytometry of the iPSC- derived MSCs stained with antibodies to surface markers CD90, CD73, CD105, CD14, CD34, and CD45, conjugated with fluorochromes PerCP, Alexa Fluor 647, PE, and APC. Isotype control is shown in green, and specific surface markers are shown in red. BMP, WNT, BMP- or WNT-induced mesoderm commitment of iPSCs.

Next, we tested whether fibroblast-like cells derived from iPSCs treated with BMP4 or CHIR possess the inherent properties of adult MSCs. Significant changes in cell morphology were observed during iPSC differentiation. Upon the induction of differentiation by activation of WNT or BMP signaling, the tightly packed cells within the iPSC colonies increased in size and gradually became spindle-like. The most noticeable differences between the cells generated in the two protocols were observed on day 7. The WNT-induced cells were relatively smaller and more rounded compared to their BMP-induced counterparts (Figure 2a). To promote MSC differentiation, we cultured iPSC-derived progenitors for 3–4 weeks in a defined MSC medium. Eventually, both the BMP- and WNT-induced cells acquired the robust MSC phenotypic characteristics with spindle-shaped morphology (Figure 2a). Then, we tested whether the MSC-like cells derived through BMP or WNT specification of the iPSCs satisfied MSC minimal criteria [37]. The MSCs derived from progenitors generated by the treatment of iPSCs with BMP 4 or CHIR possessed the ability to differentiate into adipogenic, osteogenic, and chondrogenic lineages when cultured in relevant induction mediums (Figure 2b). The iPSC-derived MSC population expressed the typical surface markers of human MSCs, including CD73, CD90, and CD105, but were negative for CD14, CD34, and CD45, as measured using flow cytometry (Figure 2c). These results suggest that iPSC specification through activation of BMP or WNT signaling within 7 days gives rise to cells that are MSC progenitors.

### 3.3. Initial BMP4 Treatment Improves Lateral Mesoderm Formation

Next, we investigated the expression profile of the WNT- and BMP-induced MSC progenitors differentiated from iPSCs. The specification of iPSCs by the manipulation of BMP4 and WNT signaling was monitored by the expression of the specific transcripts for LM, *HAND1*, *HAND2*, and *FOXF1*, and PM, *DLL1*, *MSGN1*, and *MEOX1* [16,28,29].

**Figure 3:**
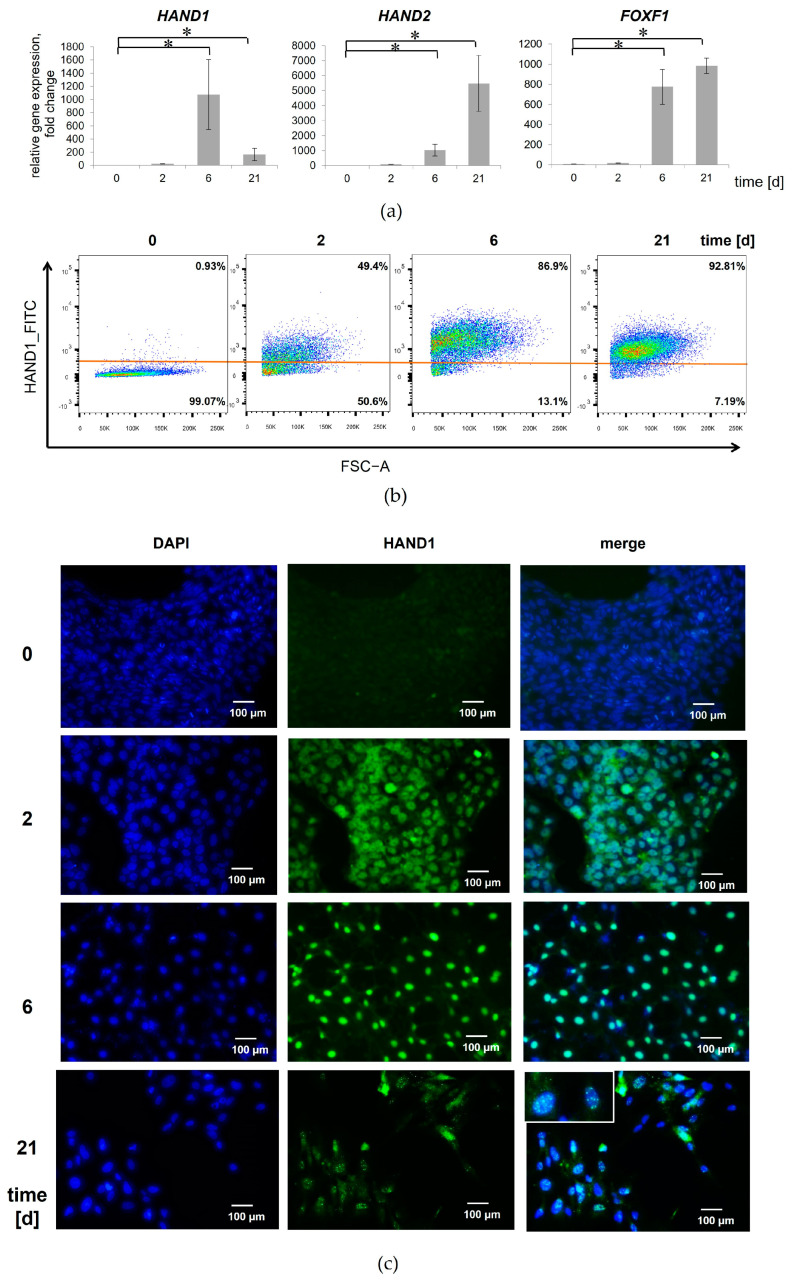
Expression of LM-specific markers during BMP-induced mesoderm specification of iPSCs. (**a**) Relative mRNA expression for *HAND1*, *HAND2*, and *FOXF1* shows increased transcript levels in the iPSCs treated with BMP4 compared with untreated iPSCs. (**b**) Flow cytometric analysis demonstrates development of HAND1+ mesenchymal lineage. (**c**) Immunostaining shows nuclear localization of HAND1 (green) in cells during mesodermal differentiation of the iPSCs. Nuclei are stained with DAPI. Scale bar, 100 μm. Data are represented as mean ± SD, *n* = 3 independent experiments. *t*-test, * reliability of differences at *p* < 0.05.

We observed that the expression level of the specific lateral plate genes, *HAND1*, *HAND2*, and *FOXF1*, increased significantly in the BMP4-treated iPSCs by day 6 of differentiation when compared to the iPSCs (Figure 3a).

In order to quantify LM differentiation efficiency, we performed the flow cytometry analysis, which showed that the expression of HAND1 in the cells gradually increased from day 2 to day 6, and by day 21, 94.8 ± 5.2% of cells were positive for HAND1. Immunofluorescence analysis also showed that HAND1 synthesis was induced in almost all the cells after the treatment of the iPSCs with BMP4 (Figure 3b). The high level of HAND1 expression was maintained during differentiation into the lateral mesoderm until the MSC stage. However, HAND1 was located in the MSCs as a distinct nuclear inclusion (Figure 3c). The flow cytometry analysis also revealed significant differences in HAND1 expression between the WNT- and BMP-induced MSC progenitors on day 6 of iPSC differentiation (Figure S1). These results suggest that cells derived from iPSCs by BMP4 specification exhibit LM characteristics within 6 days.

### 3.4. WNT Specification of iPSCs Promotes Paraxial Mesoderm Development

**Figure 4:**
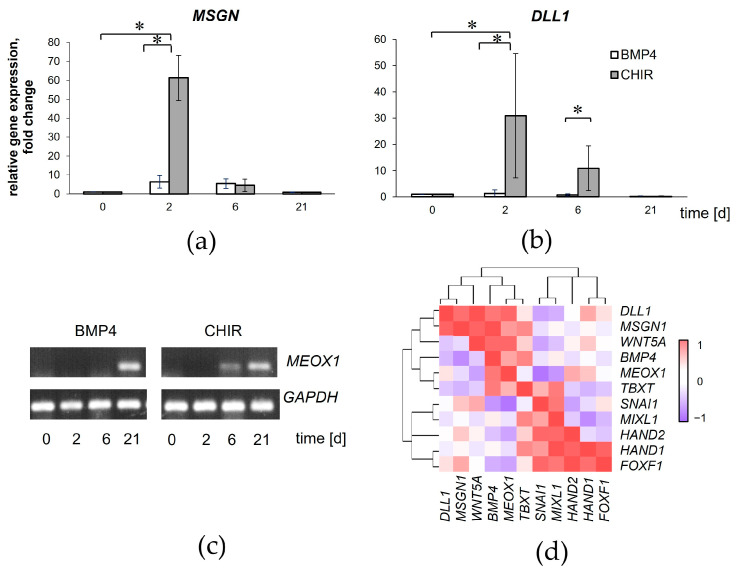
Expression of PM-specific markers during WNT-induced mesoderm specification of iPSCs. (**a**,**b**) Relative mRNA expression for *DLL1* and *MSGN1* in the iPSCs treated with CHIR and BMP4. Data are represented as mean ± SD, *n* = 3 independent experiments. * *p* < 0.05. *t*-test, * reliability of differences at *p* < 0.05. (**c**) The induction of *MEOX1* expression in the iPSCs treated with CHIR on day 6 of differentiation. (**d**) Heatmap defines meta-clusters based on Pearson correlation among LM (*HAND1*, *HAND2*, and *FOXF1*), PM (*DLL1*, *MSGN1*, and *WNT5A*), and primitive streak (*TBXT*, *SNAI1*, *MIXL1*, and *BMP4*) signal profiles. *n* = 3 independent experiments. The color scale is shown at the right of the heatmap.

The WNT-induced mesodermal differentiation of iPSCs was accompanied by an increased expression of the PM-specific markers *DLL1* and *MSGN1* within the first 6 days (Figure 4a,b). Although the level of *DLL1* mRNA expression increased in the cells, we were unable to detect DLL1 protein in the cells using immunohistochemistry and flow cytometry. After 6 days, the levels of *DLL1* and *MSGN1* transcripts decreased significantly. We also observed the induction of *MEOX1* expression in the CHIR-treated iPSCs by day 6 of differentiation. At the same time, *MEOX1* expression was not detected in the BMP-4-treated iPSCs. In addition, we showed that *MEOX1* was also expressed in the MSCs delivered through both BMP- and WNT-induced intermediate stages (Figure 4c). These results suggest that the cells derived from iPSCs by WNT specification exhibited several PM-specific characteristics within 6 days. In our experimental conditions, *MEOX1* appeared to be the factor that distinguished the WNT- and BMP-induced MSC progenitors differentiated from iPSCs on day 6 of differentiation.

The correlation analysis established low relatedness among the cell groups expressing specific mRNA for the primitive streak (*TBXT*, *SNAI1*, *MIXL1*, and *BMP4*), the LM (*HAND1*, *HAND2*, and *FOXF1*), and the PM (*DLL1*, *MSGN1*, and *WNT5A*). On the other hand, the meta-clusters and hierarchical dendrogram presented in the heatmap show a high correlation with the LM and PM signal profiles within the relevant groups (Figure 4d).

### 3.5. Cell Surface Antigen Exposing Divorces of WNT- and BMP-Induced Progenitors from MSCs

**Figure 5:**
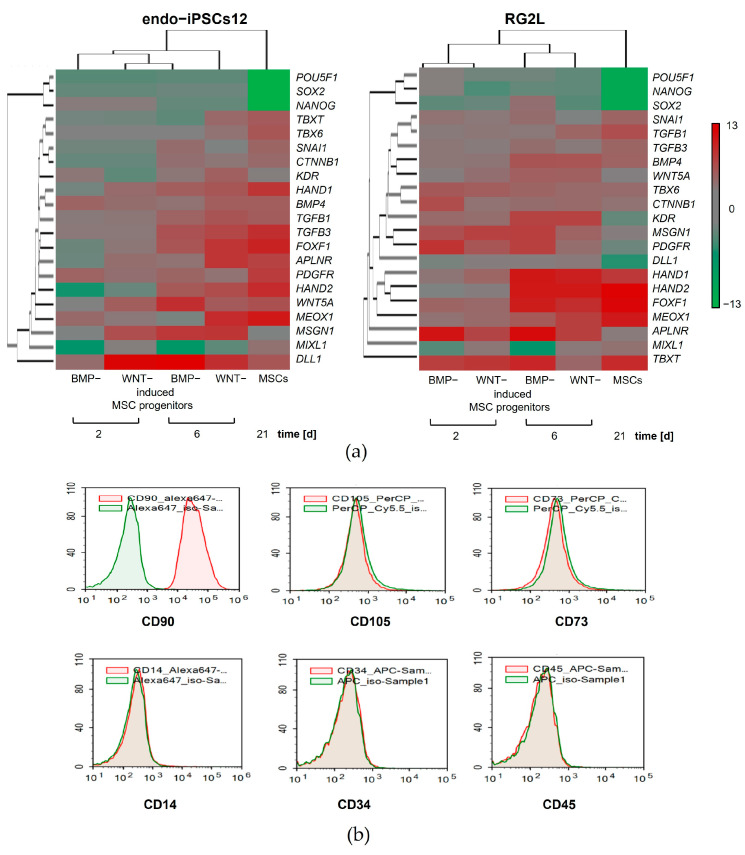
Differential gene expression and cell surface protein exposure during mesodermal specification of iPSCs. (**a**) Heatmaps showing the changes in gene signatures during BMP- or WNT-induced mesodermal specification of the endo-ips12 and RG2L iPSCs lines. The expression levels of genes are presented using fold-change values transformed to log2 format in comparison to their values in the parental iPSCs. The log2 (fold-change values) and the color scale are shown at the right of the heatmap. The housekeeping gene GAPDH was used for normalization. *n* = 3 independent experiments. (**b**) Flow cytometry of iPSC-derived MSC progenitors stained with antibodies to surface markers CD90, CD73, CD105, CD14, CD34, and CD45, conjugated with fluorochromes PerCP, Alexa Fluor 647, PE, and APC. Isotype control is shown in green, and specific surface markers are shown in red.

To understand the change in expression profiles during the mesodermal specification of iPSCs under CHIR and BMP4 exposure, transcription factors, surface markers, and growth factors were analyzed using qRT-PCR. The expression levels of 21 genes were estimated in comparison to their values in the parental iPSCs using fold-change values. The stimulation of iPSCs with BMP4 or CHIR resulted in the expected changes in the gene expression profile. The heatmaps show that the expression of the pluripotency genes *POU5F1*, *NANOG*, and *SOX2* and the primitive streak-specific genes, including *TBXT*, *MIXL1*, *TBX6*, and *SNAI1*, was downregulated during the mesodermal differentiation of the iPSCs toward the MSCs. In contrast, the expression levels of the specific transcripts for LM (*HAND1*, *HAND2*, and *FOXF1*) and PM (*DLL1* and *MSGN1*) gradually increased with differentiation into MSCs. Moreover, the observed changes in gene expression were in accordance with a continuously enhanced mesoderm specification. However, despite the general trends, different cell lines exhibited individual patterns of gene expression (Figure 5a).

Hierarchical clustering was performed to determine whether the gene expression patterns significantly distinguished the isogenic MSCs from their WNT- and BMP-induced progenitors differentiated from iPSCs. Regardless of the approach to iPSC stimulation, the WNT- and BMP-induced MSC progenitors on days 2 and 6 clustered together, respectively, according to the similarities of the gene expression signatures. At the same time, the cluster corresponding to the MSCs showed a significant difference from their isogenic progenitors (Figure 5a).

The evaluation of the expression of the surface markers *APLNR*, *PDGFRB*, and *KDR* did not reveal any significant difference between the MSCs and their WNT- and BMP-induced progenitors differentiated from the iPSCs. However, the flow cytometry analysis of the MSC surface antigens CD73, CD90, and CD105 demonstrated that on day 6 of differentiation, the WNT- and BMP-induced MSC progenitors exhibited only CD90 on the cell membranes. The markers of MSC, such as CD73 and CD105, were not exposed on the membranes of these cells. At the same time, the WNT- and BMP-induced MSC progenitors differentiated from the iPSCs also did not express the hematopoietic cell markers CD14, CD34, and CD45 (Figure 5b). Due to the lack of CD73 and CD105 expression, the WNT- and BMP-induced MSC progenitors cannot be classified as MSCs [37]. Hence, the membrane expression of CD73 and CD105 may separate MSCs from their WNT- and BMP-induced progenitors.

Our results suggest that WNT- and BMP-induced progenitors and MSCs are isogenic cell models corresponding to different stages of human development that may be derived from one parental iPSC line. These isogenic cell models can be applied for comparative investigations of the functional activity of MSCs and their progenitors in the pro-inflammatory environment.

### 3.6. Inflammatory Status of Mesodermal Progenitor Cells Differentiated from iPSCs in Resting Conditions

**Figure 6:**
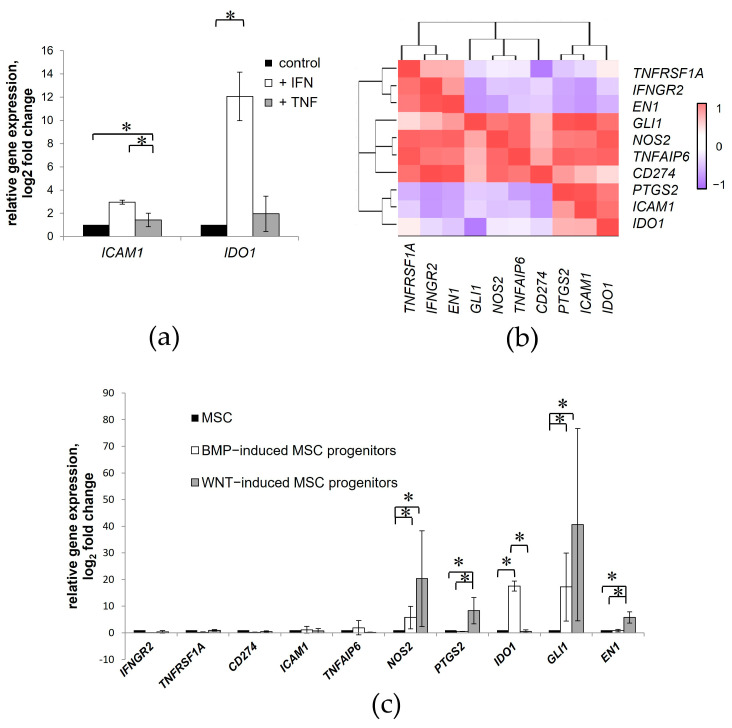
Inflammatory status of mesodermal cells differentiated from iPSCs. (**a**) Relative mRNA expression for *IDO1* and *ICAM1* in iPSC-derived MSCs after stimulation with IFN-γ and TNF-α. The housekeeping gene, GAPDH was used for normalization. (**b**) Heatmap showing Pearson correlation between gene expression of inflammatory mediators among the isogenic MSCs and WNT- and BMP-induced progenitors upon IFN-γ and TNF-α treatment. (**c**) Basal levels of inflammatory response-related gene expression in the WNT- and BMP-induced MSC progenitors compared to their isogenic MSCs. Data are represented as mean ± SD, *n* = 3 independent experiments. *t*-test, * reliability of differences at *p* < 0.05.

MSCs are known to secrete a variety of cytokines, chemokines, and growth factors, some of which change in response to local environmental cues and stimulate tissue regeneration. However, the known mechanisms underlying the immunomodulatory activity of adult MSCs cannot perfectly describe the complex and multifactorial processes that support the successful healing of embryonic tissues. To gain more insights into the mechanisms underlying complete embryonic regeneration, we compared the ability of iPSC-derived MSC progenitors to respond to the proinflammatory signals with their isogenic MSCs. The proinflammatory microenvironment was previously found to alter the functional status of adult MSCs through the upregulation of cyclooxygenase-2 (COX-2, *PTGS2*), indoleamine 2,3-dioxygenase (IDO, *IDO1*), inducible nitric oxide synthase (iNOS, *NOS2*), transforming growth factor beta (TGF-β, *TGFB*), galectins, programmed cell death 1 ligand 1 (PD-L1, *CD274*), ICAM-1, etc. [38]. In particular, IFN-γ stimulation affected MSC immunosuppressive activity through the induction of the IDO enzyme, which catabolizes tryptophan into kynurenine [39,40]. Previously, we showed that the most prominent immunosuppressive activity of adult MSCs was observed after 48 h when exposed to proinflammatory factors [26]. Considering the results of the previous studies, we assessed here the expression of inflammatory mediator genes in isogenic MSCs and their WNT- and BMP-induced progenitors differentiated from iPSCs after 48-h stimulation with 20 ng/mL IFN-γ or TNF-α. To evaluate the functional activity of cells in a proinflammatory microenvironment, we used 6-day-old iPSC-derived progenitors since they had a gene expression profile comparable to that of MSCs, while 2-day-old progenitors were more similar to the parental iPSCs (Figure 5a). Here, we observed that iPSC-derived MSCs possess a similar ability to respond to IFN-γ treatment with the induction of *IDO1* expression and increased levels of *ICAM1* relative to adult MSCs (Figure 6a). The stimulation of MSCs with TNFα also induces several synergistic and overlapping functionalities with integrative effects through activation of the NF-κB pathway, which regulates the expression of nearly 400 genes, including the iNOS and COX-2 enzymes [41]. Under our experimental conditions, the gene expression correlation analysis showed a low pairwise relationship between the inflammatory mediators that were expressed in response to IFN-γ or TNF-α in MSCs and their isogenic WNT- and BMP-induced progenitors (Figure 6b).

The qPCR analysis of the intact cells revealed that WNT- and BMP-induced MSC progenitors constitutively secrete higher levels of several inflammatory mediators compared to their isogenic MSCs. Expression of *NOS2* and transcription factor *GLI1* was significantly higher in both the WNT- and BMP-induced MSC progenitors than in MSCs. The WNT-induced MSC progenitors exhibited higher expression of *PTGS2* and *EN1* compared with the MSCs and BMP-induced counterparts, whereas the BMP-induced MSC progenitors initially expressed enhanced levels of *IDO1* (Figure 6c). Our results demonstrate that CHIR and BMP priming of iPSCs not only determines the specification of MSC progenitors but also influences their initial inflammatory status.

### 3.7. WNT- and BMP-Induced MSC Progenitors Differetiated from iPSCs Exhibit Reduced Response to Inflammatory Signals Compared to MSCs

Next, we compared the expression of inflammatory response-related genes between the isogenic MSCs and WNT- and BMP-induced progenitors in response to IFN-γ and TNF-α treatment.

**Figure 7:**
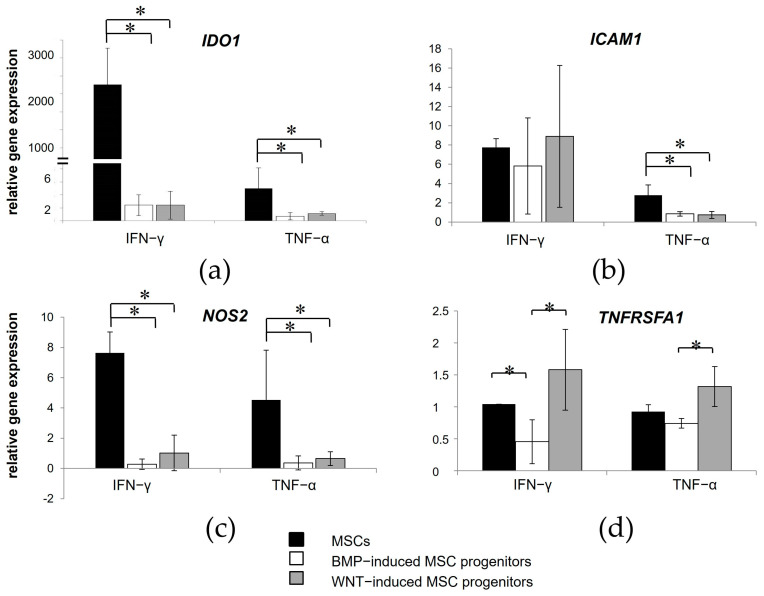
Relative mRNA expression of *IDO1* (**a**), *ICAM1* (**b**), *NOS2* (**c**), and *TNFRSFA1* (**d**) in the BMP- and WNT-induced MSC progenitors and the MSCs differentiated from iPSC after IFN-γ and TNF-α treatment. Data are normalized to basal expression levels in the same cell lines without stimulation. Data are represented as mean ± SD, *n* = 3 independent experiments. *t*-test, * reliability of differences at *p* < 0.05.

Although the WNT- and BMP-induced MSC progenitors exhibited enhanced mRNA expression of several proinflammatory mediators in resting conditions, they demonstrated a decreased response to inflammatory signals from the microenvironment when compared to the isogenic MSCs. We detected reduced expression levels of *IDO1* and *NOS2* in the WNT- and BMP-induced MSC progenitors relative to MSCs in response to stimulation with IFN-γ and TNF-α (Figure 7a,c). The expression levels of *ICAM1* were comparable between all groups when exposed to IFN-γ but showed a decrease in the WNT- and BMP-induced MSC progenitors after stimulation with TNF-α (Figure 7b). The BMP-induced MSC progenitors showed a decrease in *TNFRSFA1* expression compared to the MSCs in response to IFN-γ application. However, *TNFRSFA1* expression varied significantly between the BMP- and WNT-induced MSC progenitors in response to both IFN-γ and TNF-α (Figure 7d).

No significant differences in the expression of *PTGS2*, TNF alpha-induced protein (TSG-6, *TNFA1P6*), *CD274*, *EN-1*, and *GLI1* were found between the isogenic WNT- and BMP-induced MSC progenitors and MSCs after stimulation with IFN-γ and TNF-α.

### 3.8. Inflammatory Response-Related Gene Expression Profiles of Isogenic Mesodermal Derivatives Differentiated from iPSCs

To gain further insights into differences in inflammatory responses between the isogenic MSCs and WNT- and BMP-induced progenitors differentiated from iPSCs, the inflammatory response-related gene expression profiles were investigated.

**Figure 8:**
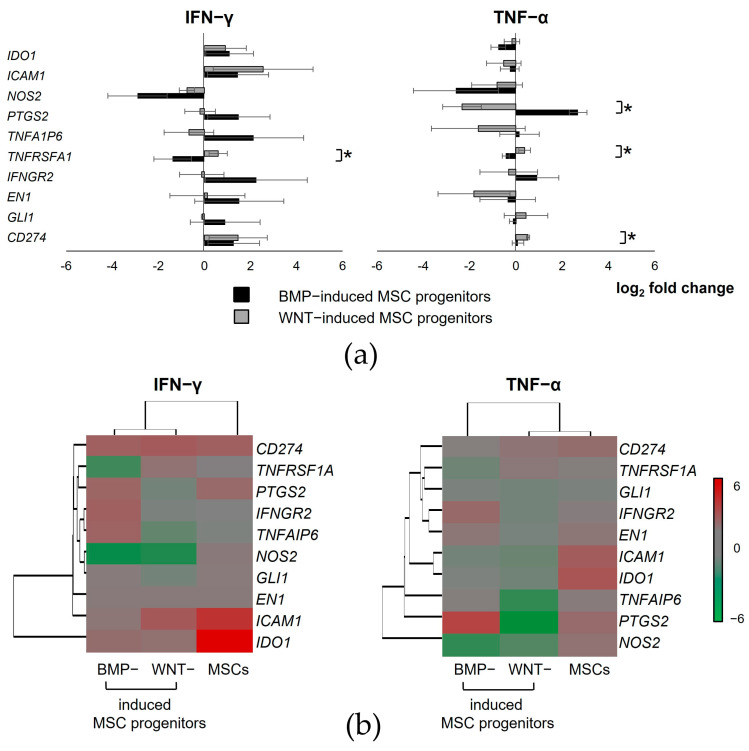
Inflammatory response-related gene signatures of mesodermal derivatives differentiated from iPSCs treated with IFN-γ and TNF-α. (**a**) Bar plots showing the diversities in inflammatory response between the WNT- and BMP-induced MSC progenitors upon stimulation with IFN-γ and TNF-α. Data are represented as mean ± SD, *n* = 3 independent experiments. *t*-test, * reliability of differences at *p* < 0.05. (**b**) Heatmaps of differentially expressed genes in the MSCs and their WNT- and BMP-induced progenitors in response to proinflammatory stimuli. The expression levels of genes are presented using fold-change values transformed to log2 format in comparison to basal expression levels in the same cell lines without stimulation. The log2 (fold-change values) and the color scale are shown at the right of the heatmap.

The most pronounced difference between the WNT- and BMP-induced MSC progenitors was observed in the expression *of PTGS2*, *TNFRSFA1*, and *CD274* upon TNF-α treatment. The BMP-induced MSC progenitors exhibited increased *PTGS2* expression. In contrast, the PTGS2 expression was downregulated in the WNT-induced MSC progenitors under the same conditions. At the same time, the difference in the expression of *PTGS2* was not statistically significant between the WNT- and BMP-induced MSC progenitors in response to stimulation with IFN-γ. *TNFRSFA1* expression was upregulated in the WNT-induced MSC progenitors but downregulated in the BMP-induced MSC progenitors upon both IFN-γ and TNF-α treatment. Stimulation with IFN-γ and TNF-α resulted in the increased expression of *CD274* in both the WNT- and BMP-induced MSC progenitors. However, the difference in *CD274* expression was statistically significant only after TNF-α application (Figure 8a). In addition, these cells showed opposite responses in the expression of *TNFA1P6*, *INFGR2*, *EN-1*, and *GLI1* in response to inflammatory stimuli. However, in this case, the differences between the WNT- and BMP-induced MSC progenitors were not statistically significant. On the other hand, the WNT- and BMP-induced MSC progenitors responded similarly to IFN-γ treatment, increasing *IDO1* and *ICAM1* expression. Conversely, the expression of these genes was downregulated in both types of cells when exposed to TNF-α. Stimulation with IFN-γ and TNF-α resulted in decreased *NOS2* expression in both the WNT- and BMP-induced MSC progenitors.

Although the WNT- and BMP-induced MSC progenitors showed substantially divergent gene expression patterns under proinflammatory conditions, a hierarchical clustering analysis revealed greater similarity between them upon IFN-γ treatment, whereas their expression profiles were distinct from those observed in the MSCs. At the same time, the MSCs were clustered together with the WNT-induced MSC progenitors upon treatment with TNF-α. Together, they showed significant diversity compared to the cluster corresponding to the BMP-induced MSC progenitors (Figure 8b).

Our results demonstrate that the WNT- and BMP-induced specification of iPSCs not only determines the initial inflammatory status of the MSC progenitors but also modulates their response to inflammatory stimuli.

### 3.9. Inflammatory Factors Influence Cell Migration

In order to assess the functional activity of the isogenic mesodermal derivatives differentiated from iPSCs in the proinflammatory microenvironment, a wound scratch assay was performed. The migration rate was compared between the WNT- and BMP-induced MSC progenitors and their isogenic MSCs upon the addition of TNFα or IFN-γ. The cells in the medium alone were used as a control. The scratch size was initially set to be 100%, and wideness was measured at different time points.

**Figure 9:**
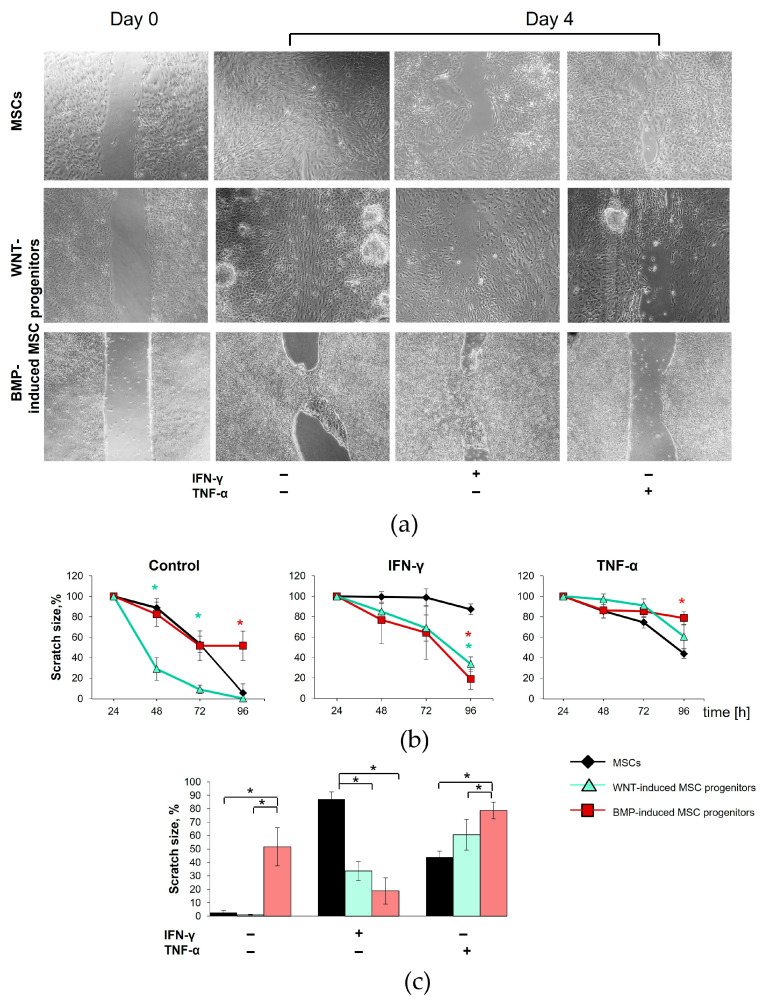
Effects of inflammatory factors on migration of mesodermal cells differentiated from iPSCs in scratch wound healing assay. (**a**) Representative images demonstrate the differences in migratory activity between the isogenic MSCs and WNT- and BMP-induced MSC progenitors under intact conditions and upon the TNFα or IFN-γ treatment. Brightfield microscopy, magnification—40×. (**b**) Time-dependent quantification of % confluency in the scratch wound area. (**c**) Changes in the size of the wounded areas 96 hours after scratching. Data are represented as mean ± SD, measured in four representative images from each sample. *n* = 3 independent experiments *t*-test, * reliability of differences at *p* < 0.05.

We found that the intact isogenic mesodermal cells derived from the iPSCs displayed distinct and heterogenic effects on wound scratch closure (Figure 9a). The WNT-induced MSC progenitors possessed the highest migration rate when compared to BMP-induced MSC progenitors and MSCs (Figure 9b). However, the scratch disappeared by the fourth day for the WNT-induced MSC progenitors and their isogenic MSCs. At this time point, the scratch of the BMP-induced MSC progenitors had not closed completely. Surprisingly, the cell arrangement in the closed scratch varied dramatically. The fibroblast-like cells of the MSCs and BMP-induced MSC progenitors were oriented in a random fashion within a closed scratch. In contrast, the cells of the WNT-induced MSC progenitors lined up in a straight manner along the scratch (Figure 9a).

The application of IFN-γ or TNF-α to the MSCs and their isogenic WNT- and BMP-induced progenitors significantly inhibited cell migration, resulting in a slower reduction in scratch size compared to the control (Figure 9b). However, the migratory capacity in response to the inflammatory signals significantly differed between the MSCs and their progenitors. Enhanced migration and more rapid scratch closure were observed in the WNT- and BMP-induced MSC progenitors compared to their isogenic MSCs upon IFN-γ treatment. In contrast, the WNT- and BMP-induced MSC progenitors displayed a lower migratory activity as compared to the MSCs in the presence of TNF-α (Figure 9c).

A significant difference in migratory activity was also found in WNT- and BMP-induced MSC progenitors. Initially, the WNT-induced MSC progenitors possessed dramatically higher migration rates compared to their BMP-induced counterparts. Inflammatory cytokines IFN-γ or TNF-α significantly decreased the migratory activity of the WNT-induced MSC progenitors compared to the baseline level. Unlike the WNT-induced MSC progenitors, IFN-γ had virtually no effect on the migration rate of the BMP-induced MSC progenitors. At the same time, migration was almost completely suppressed in the BMP-induced MSC progenitors upon TNF-α treatment (Figure 9a–c).

Notably, our results on the migration activity of isogenic mesodermal cells in a proinflammatory microenvironment are consistent with the hierarchical clustering analysis of inflammatory gene signatures described in the previous section. The WNT- and BMP-induced MSC progenitors showed similar migratory activity in response to IFN-γ. However, they both significantly differed from the MSCs under the same conditions. This result is consistent with the greater proximity in inflammatory response-related gene expression signatures between the WNT- and BMP-induced MSC progenitors compared to MSCs (Figure 8b). At the same time, the BMP-induced MSC progenitors showed significant diversity compared to MSCs and WNT-induced MSC progenitors in gene expression signatures upon TNF-α treatment. The same effect was observed in the scratch test (Figure 8b and Figure 9b,c).

Our results indicate that the initial activation of WNT/β-catenin or BMP signaling pathways can guide not only the mesodermal specification of iPSCs but also determine functional heterogeneity of the WNT- and BMP-induced MSC progenitors in the proinflammatory microenvironment. The strong difference in the initial proinflammatory status and behavior upon IFN-γ or TNF-α treatment of the WNT- and BMP-induced MSC progenitors and MSCs suggests that at least two alternative mechanisms may be involved in the regulation of fetal tissue regeneration compared to those in adults.

## 4. Discussion

The scarless healing of fetal tissues is assumed to occur with the contribution of fetal MSCs (mesodermal progenitors), which phenotypically and functionally differ from those in adults. However, the differences in the molecular mechanisms involving soluble factors and adhesive and receptor-dependent intercellular communications, which determine the functional activity of mesodermal cells in damaged tissues in fetuses and adults, remain virtually unknown. Thus, the generation and description of the properties of mesodermal cell models corresponding to different stages of human development are necessary to ensure the subsequent comparative studies of their functional activity in vitro.

It was previously shown that WNT and/or BMP signaling pathways play a crucial role in mesodermal specification and navigation of human PSCs toward LM and PM [16,27,28,29,30,31,32,33,34]. Specification into lateral mesoderm cells is determined by the expression of *HAND1* and *FOXF1*. Paraxial mesoderm cells are defined by the expression of *DLL3* and *MSGN1* [16,28,29,30]. Recently, we found that the deprivation of pluripotency-supporting medium combined with bFGF and activin A coexposure was suitable for the releasing of iPSCs from the pluripotency state and the triggering of the commitment to the primitive streak in the first 24 h of differentiation [27]. Here, we demonstrated that a short initial CHIR pulse that activated WNT signaling was sufficient to induce the primitive streak cells derived from iPSCs to differentiate into cells phenotypically referred to as human embryonic PM cells and to maintain strong effects over a week, although nuclear β-catenin gradually declined to the control level. The pulse treatment of the cells with CHIR resulted in a transient increase in the expression of the paraxial mesoderm-specific genes *DLL1* and *MSGN1* and the induction of the early somite marker *MEOX1*. Hence, initial WNT activation via GSK3β inhibition can induce the commitment of iPSCs to PM, after which external WNT signals become dispensable. Our data are in agreement with the well-described WNT-induced formation of paraxial mesoderm and downstream lineages (presomitic and somitic mesoderm) in vitro from human ESCs and iPSCs [16,28,31,32,33].

Several efficient protocols have been described for obtaining cells with the characteristics of LM from human hPSCs using varying combinations of FGF2, BMP4, the ROCK inhibitor Y27632, and follistatin. hPSC-derived LM cells were shown to express the *CDX2*, *TBX3*, *FOXF1*, *IRX3*, *BMP4*, *WNT5A*, *HAND1*, and *HAND2* genes, which are representative of the lateral plate/extraembryonic mesoderm [16,29,30,34,42,43]. In our experimental conditions, the exposure of precommitted iPSCs to BMP4 elicited an increased expression of *HAND1*, *HAND2*, and *FOXF1* compared to the iPSCs treated with CHIR. The sustained expression of *HAND1* during iPSC differentiation toward MSCs was also demonstrated with immunohistochemistry and flow cytometry analysis, confirming that we successfully established an efficient protocol for the induction of iPSC differentiation into LM cells.

However, we found that the mesodermal specification of iPSCs by CHIR or BMP4 resulted in overlapping induction of LM- and PM-specific gene expression, although the correlation coefficients demonstrated weak relationships between the groups. These observations support an intricate interplay between WNT/β-catenin and BMP receptor signaling in the mesodermal progenitors described in previous studies [44,45,46].

We found that WNT-induced mesoderm cells, like their BMP-induced counterparts, were able to give rise to cells corresponding to adult human MSCs in the expression of the surface markers CD105, CD90, and CD73; in the absence of expression of the hematopoietic cell markers CD45, CD34, and CD14; and in the ability to undergo adipogenic, chondrogenic, and osteogenic differentiation. Our data are in accordance with those of a large body of literature supporting the exhibition of typical surface markers, differentiation potential, and immunomodulatory properties in hPSC-derived MSCs [20,21,28,29,34,47]. Thus, our observations confirm that the WNT- and BMP-induced MSC progenitors obtained via differentiation from iPSCs in our experimental conditions are the progenitors of MSCs. Importantly, both cell types during further cultivation were positive for CD90 but did not exhibit CD73 or CD105 on the cell membranes. The MSC progenitors differentiated from the iPSCs also did not express the hematopoietic cell markers CD14, CD34, and CD45, like their subsequent isogenic MSCs. The partial discrepancy in the expression of surface markers by the MSC progenitors differentiated from hPSCs between our data and those of others may be explained by differences in the experimental protocols [21,42].

To advance our knowledge and understanding of the processes of fetal wound healing and scarring, we performed, for the first time, a comparative analysis of the functional activity of isogenic MSCs and their progenitors differentiated from iPSCs in a proinflammatory microenvironment. Adult MSCs are known to possess unique immunomodulatory activity. The cytokines, chemokines, growth factors, and extracellular vesicles secreted by MSCs are involved in the regulation of intracellular signaling cascades that stimulate angiogenesis, the recruitment of cells to sites of inflammation, and remodeling of the extracellular matrix [10,18,48,49]. Our previous findings revealed that adult MSC immunomodulatory activity in response to the proinflammatory microenvironment generated by activated peripheral blood mononuclear cells in co-culture was exerted through the induction of IDO and the increase in ICAM-1 levels, which are involved in the regulation of IDO synthesis in MSCs [26,50,51]. In the present study, we found that iPSC-derived MSCs exhibited similar activity in response to IFN-γ or TNF-α treatment compared to adult MSCs (Figure 6a).

The functional activity of WNT- and BMP-induced MSC progenitors differentiated from iPSCs in a proinflammatory microenvironment has not yet been described. Our results indicate that MSC progenitors differentiated from iPSCs constitutively secrete higher levels of several inflammatory mediators when compared to their isogenic MSCs in resting cultures. We observed that the MSC progenitors derived from iPSCs through the activation of WNT signaling possessed higher expression levels of *PTGS2* and *EN1*. When the BMP signaling pathway was activated in the iPSCs, the resulting MSC progenitors had a higher level of *IDO1* expression. Our results also showed that the expression levels of transcriptional factor *GLI1* were significantly higher in both the WNT- and BMP-induced MSC progenitors compared to isogenic MSCs. This observation confirms previous data demonstrating that Sonic Hedgehog (SHH) signal activation can trigger embryonic stem cells into the healing process of postnatal skin wounds in mice, promoting their proliferation and migration to the wound area [52,53,54]. However, we found here that the response of WNT- and BMP-induced MSC progenitors to the proinflammatory microenvironment generated by IFN-γ or TNF-α treatment was significantly less pronounced compared to the isogenic MSCs. We detected significantly lower expression levels of *IDO1*, *NOS2*, and *ICAM1* in both the WNT- and BMP-induced MSC progenitors than in the isogenic MSCs after IFN-γ and TNF-α treatment. The expression levels of *PTGS2*, *TNFRSF1A*, *TNFAIP6*, *INFGR2*, *EN-1*, and *GLI1* varied ambivalently between the WNT- and BMP-induced MSC progenitors under the same conditions. The BMP-induced MSC progenitors differed significantly from their WNT-induced counterparts in the expression of *PTGS2*, *TNFRSF1A*, and *CD274* upon stimulation with TNF-α.

Several studies have reported that hPSC-derived MSCs possess improved therapeutic efficacy when compared to tissue-specific MSCs in different animal models of diseases [20,55,56,57]. Interestingly, in a mouse model of skin wounds, MSCs derived from hPSCs through LM accelerated wound healing faster than MSCs of paraxial origin. In contrast, treatment with MSCs originating from PM progenitors more effectively cured pressure ulcers than treatment with LM-derived MSCs [47]. To evaluate the potential activity of MSC progenitors in wound healing, we performed a scratch assay in vitro. Our results revealed that WNT-induced MSC progenitors had the highest migratory ability and rapidly closed the scratches. In the case of the BMP-induced MSC progenitors, the scratch closure occurred much more slowly compared to their WNT-induced counterparts and isogenic MSCs. Surprisingly, within the closed scratch, the patterns of cell arrangement varied greatly. The WNT-induced MSC progenitors were arranged in a straight manner along the scratch, whereas the MSCs and BMP-induced MSC progenitors were oriented in a random manner. Under proinflammatory conditions, the migratory activity was reduced in all the cell types compared to the background controls. However, the WNT- and BMP-induced MSC progenitors possessed higher migratory activity compared to the isogenic MSCs when treated with IFN-γ. Such functional plasticity can be explained by the initial phenotypic status of WNT- and BMP-induced MSC progenitors and MSCs. For example, the WNT-induced MSC progenitors had higher expression levels of EN1 compared to the MSCs and BMP-induced MSC progenitors. Several studies revealed that embryonic cells that have En1 are responsible for the bulk of connective tissue deposition during embryonic development and wound healing and contribute to scarring in various models of wounds. Conversely, En1-negative fibroblasts were shown to drive dermal development and regeneration; furthermore, they do not participate in scar production [58,59]. Moreover, preventing En1 activation in fibroblasts resulted in a more reticular lattice arrangement of ECM and enabled wound regeneration without scarring in mice [60]. On the other hand, increased IDO activity is thought to be a negative regulator of the inflammatory responses, as it keeps proinflammatory signaling in check [61,62]. Thus, the initially elevated IDO levels may contribute to the reduced response of BMP-induced MSC progenitors to IFN-γ and allow faster scratch closure compared to MSCs. Increased basal levels of GLI1 in WNT- and BMP-induced MSC progenitors may also determine some of the functional features that distinguish them from isogenic MSCs, confirming the essential role of SHH signaling in normal wound healing [52,53,54]. On the other hand, the BMP-induced MSC progenitors exhibited significantly higher *PTGS2* expression levels and the lowest migratory activity compared with the WNT-induced MSC progenitors and MSCs when treated with TNF-α. Wilgus et al. demonstrated differential expression of the COX-2 enzyme in a murine model of early and late gestation fetal wounds with scarless and fibrotic repair. Basal keratinocytes, inflammatory cells, and stromal cells were shown to express COX-2 protein in skin wounds made at embryonic day 18 (E18) but not in wounds made at E15 [63]. These observations confirm that not only the initial inflammatory status but also functional heterogeneity of the WNT- and BMP-induced MSC progenitors may determine their behavior in the proinflammatory microenvironment.

Taken together, our results confirmed that important signaling pathways, such as WNT, BMP, FGF, and SHH, which play critical roles in tissue patterning during embryonic development and maintaining tissue homeostasis in adults, may also be involved in mechanisms that determine successful healing without scarring. Other conserved signaling pathways such as Notch, TGF-β, and EGF may also be involved in the regulation of cell fate decisions and differentiation during embryonic and postnatal development [64,65]. Crosstalk between these signaling pathways might have a role in aging-related tissue fibrosis [66,67,68]. However, the role of these pathways in the processes of mesodermal differentiation of pluripotent cells remains poorly understood.

Indeed, our study has certain limitations and potential weaknesses since we focused on stimulation with individual inhibitors, growth factors, or proinflammatory cytokines, although the combination of the various factors has crucial importance in the dynamic response of cells during human embryonic development and in the inflammatory milieu. However, we believe that a further analysis of functional activity and gene/protein expression in isogenic mesodermal cells of various stages of human development differentiated from iPSCs under the receptor-dependent activation of signaling pathways will reveal key molecules and therapeutic targets that determine perfect healing.

In conclusion, the results presented here show that WNT/β-catenin or BMP activation at the initiation of iPSC differentiation is a key step towards the exit from pluripotency, the enhancement of mesoderm commitment, and the specification of PM and LM, through which MSCs can be generated. These isogenic cell models corresponding to the various stages of human development, generated by the sequential, multistage, and specifically targeted mesodermal differentiation of iPSCs could be beneficial for the elucidation of the biological characteristics and functional state of their in vivo counterparts in an inflammatory microenvironment; thus, new opportunities are presented to determine the mechanisms underlying the complete regenerative phenomenon and the design of new clinical approaches for regenerative medicine.

## Data Availability

Dataset available on request from the authors.

## Supplementary Materials

The following supporting information can be downloaded at: https://www.mdpi.com/article/10.3390/cells13211820/s1, Table S1: Human-specific oligonucleotide primers; Table S2: Antibodies and their final dilutions used for immunofluorescence analysis and flow cytometry. Figure S1: Cross-activation of HAND1 expression in iPSCs treated with CHIR and BMP4.

## References

[1] Shaw T.J., Martin P. (2009). Wound repair at a glance. J. Cell Sci..

[2] Marshall C.D., Hu M.S., Leavitt T., Barnes L.A., Lorenz H.P., Longaker M.T. (2018). Cutaneous Scarring: Basic Science, Current Treatments, and Future Directions. Adv. Wound Care.

[3] Furman D., Campisi J., Verdin E., Carrera-Bastos P., Targ S., Franceschi C., Ferrucci L., Gilroy D.W., Fasano A., Miller G.W. (2019). Chronic inflammation in the etiology of disease across the life span. Nat. Med..

[4] Kolimi P., Narala S., Nyavanandi D., Youssef A.A.A., Dudhipala N. (2022). Innovative Treatment Strategies to Accelerate Wound Healing: Trajectory and Recent Advancements. Cells.

[5] Kim E.Y., Hussain A., Khachemoune A. (2023). Evidence-based management of keloids and hypertrophic scars in dermatology. Arch. Dermatol. Res..

[6] Colwell A.S., Longaker M.T., Lorenz H.P. (2005). Mammalian fetal organ regeneration. Adv. Biochem. Eng. Biotechnol..

[7] Moore A.L., Marshall C.D., Barnes L.A., Murphy M.P., Ransom R.C., Longaker M.T. (2018). Scarless wound healing: Transitioning from fetal research to regenerative healing. Wiley Interdiscip. Rev. Dev. Biol..

[8] Thomson J.A., Itskovitz-Eldor J., Shapiro S.S., Waknitz M.A., Swiergiel J.J., Marshall V.S., Jones J.M. (1998). Embryonic stem cell lines derived from human blastocysts. Science.

[9] Takahashi K., Tanabe K., Ohnuki M., Narita M., Ichisaka T., Tomoda K., Yamanaka S. (2007). Induction of pluripotent stem cells from adult human fibroblasts by defined factors. Cell.

[10] Pittenger M.F., Discher D.E., Péault B.M., Phinney D.G., Hare J.M., Caplan A.I. (2019). Mesenchymal stem cell perspective: Cell biology to clinical progress. NPJ Regen. Med..

[11] Han Y., Yang J., Fang J., Zhou Y., Candi E., Wang J., Hua D., Shao C., Shi Y. (2022). The secretion profile of mesenchymal stem cells and potential applications in treating human diseases. Signal Transduct. Target. Ther..

[12] Sheng G. (2015). The developmental basis of mesenchymal stem/stromal cells (MSCs). BMC Dev. Biol..

[13] Kimelman D. (2006). Mesoderm induction: From caps to chips. Nat. Rev. Genet..

[14] Chhabra S., Liu L., Goh R., Kong X., Warmflash A. (2019). Dissecting the dynamics of signaling events in the BMP, WNT, and NODAL cascade during self-organized fate patterning in human gastruloids. PLoS Biol..

[15] Teague S., Primavera G., Chen B., Liu Z.Y., Yao L., Freeburne E., Khan H., Jo K., Johnson C., Heemskerk I. (2024). Time-integrated BMP signaling determines fate in a stem cell model for early human development. Nat. Commun..

[16] Loh K.M., Chen A., Koh P.W., Deng T.Z., Sinha R., Tsai J.M., Barkal A.A., Shen K.Y., Jain R., Morganti R.M. (2016). Mapping the Pairwise Choices Leading from Pluripotency to Human Bone, Heart, and Other Mesoderm Cell Types. Cell.

[17] Weiss A.R.R., Dahlke M.H. (2019). Immunomodulation by Mesenchymal Stem Cells (MSCs): Mechanisms of Action of Living, Apoptotic, and Dead MSCs. Front. Immunol..

[18] Prockop D.J. (2013). Concise review: Two negative feedback loops place mesenchymal stem/stromal cells at the center of early regulators of inflammation. Stem Cells.

[19] Wang T., Zhang J., Liao J., Zhang F., Zhou G. (2020). Donor genetic backgrounds contribute to the functional heterogeneity of stem cells and clinical outcomes. Stem Cells Transl. Med..

[20] Wang H., Li D., Zhai Z., Zhang X., Huang W., Chen X., Huang L., Liu H., Sun J., Zou Z. (2019). Characterization and Therapeutic Application of Mesenchymal Stem Cells with Neuromesodermal Origin from Human Pluripotent Stem Cells. Theranostics.

[21] Kimbrel E.A., Kouris N.A., Yavanian G.J., Chu J., Qin Y., Chan A., Singh R.P., McCurdy D., Gordon L., Levinson R.D. (2014). Mesenchymal stem cell population derived from human pluripotent stem cells displays potent immunomodulatory and therapeutic properties. Stem Cells Dev..

[22] Wang X., Lazorchak A.S., Song L., Li E., Zhang Z., Jiang B., Xu R.H. (2016). Immune modulatory mesenchymal stem cells derived from human embryonic stem cells through a trophoblast-like stage. Stem Cells.

[23] Suzdaltseva Y., Kiselev S.L. (2023). Mesodermal Derivatives of Pluripotent Stem Cells Route to Scarless Healing. Int. J. Mol. Sci..

[24] Lagarkova M.A., Shutova M.V., Bogomazova A.N., Vassina E.M., Glazov E.A., Zhang P., Rizvanov A.A., Chestkov I.V., Kiselev S.L. (2010). Induction of pluripotency in human endothelial cells resets epigenetic profile on genome scale. Cell Cycle.

[25] Novosadova E.V., Nekrasov E.D., Chestkov I., Surdina A.V., Vasina E.M., Bogomazova A.N., Manuilova E.S., Arsenyeva E.L., Simonova V.V., Konovalova E.V. (2016). A platform for studying molecular and cellular mechanisms of Parkinson s disease based on human induced pluripotent stem cells. Sovrem. Tehnol. Med..

[26] Suzdaltseva Y., Goryunov K., Silina E., Manturova N., Stupin V., Kiselev S.L. (2022). Equilibrium among Inflammatory Factors Determines Human MSC-Mediated Immunosuppressive Effect. Cells.

[27] Selezneva A.V., Korobko E.V., Kiselev S.L., Suzdaltseva Yu G. (2024). Expression profile of isogenic early mesodermal cells differentiated from human induced pluripotent stem cells. J. Evol. Biochem. Physiol..

[28] Nakajima T., Shibata M., Nishio M., Nagata S., Alev C., Sakurai H., Toguchida J., Ikeya M. (2018). Modeling human somite development and fibrodysplasia ossificans progressiva with induced pluripotent stem cells. Development.

[29] Wei Y., Wang B., Jia L., Huang W., Xiang A.P., Fang C., Liang X., Li W. (2022). Lateral Mesoderm-Derived Mesenchymal Stem Cells with Robust Osteochondrogenic Potential and Hematopoiesis-Supporting Ability. Front. Mol. Biosci..

[30] Smith C.A., Humphreys P.A., Naven M.A., Woods S., Mancini F.E., O’Flaherty J., Meng Q.J., Kimber S.J. (2023). Directed differentiation of hPSCs through a simplified lateral plate mesoderm protocol for generation of articular cartilage progenitors. PLoS ONE.

[31] Kreuser U., Buchert J., Haase A., Richter W., Diederichs S. (2020). Initial WNT/beta-Catenin Activation Enhanced Mesoderm Commitment, Extracellular Matrix Expression, Cell Aggregation and Cartilage Tissue Yield from Induced Pluripotent Stem Cells. Front. Cell Dev. Biol..

[32] Umeda K., Zhao J., Simmons P., Stanley E., Elefanty A., Nakayama N. (2012). Human chondrogenic paraxial mesoderm, directed specification and prospective isolation from pluripotent stem cells. Sci. Rep..

[33] Xi H., Fujiwara W., Gonzalez K., Jan M., Liebscher S., Van Handel B., Schenke-Layland K., Pyle A.D. (2017). In Vivo Human Somitogenesis Guides Somite Development from hPSCs. Cell Rep..

[34] Liu T.M., Yildirim E.D., Li P., Fang H.T., Denslin V., Kumar V., Loh Y.H., Lee E.H., Cool S.M., Teh B.T. (2020). Ascorbate and Iron Are Required for the Specification and Long-Term Self-Renewal of Human Skeletal Mesenchymal Stromal Cells. Stem Cell Rep..

[35] Yamaguchi T.P., Bradley A., McMahon A.P., Jones S. (1999). A Wnt5a pathway underlies outgrowth of multiple structures in the vertebrate embryo. Development.

[36] Andre P., Song H., Kim W., Kispert A., Yang Y. (2015). Wnt5a and Wnt11 regulate mammalian anterior-posterior axis elongation. Development.

[37] Dominici M., Le Blanc K., Mueller I., Slaper-Cortenbach I., Marini F., Krause D., Deans R., Keating A., Prockop D., Horwitz E. (2006). Minimal criteria for defining multipotent mesenchymal stromal cells. The International Society for Cellular Therapy position statement. Cytotherapy.

[38] Shi Y., Wang Y., Li Q., Liu K., Hou J., Shao C., Wang Y. (2018). Immunoregulatory mechanisms of mesenchymal stem and stromal cells in inflammatory diseases. Nat. Rev. Nephrol..

[39] DelaRosa O., Lombardo E., Beraza A., Mancheño-Corvo P., Ramirez C., Menta R., Rico L., Camarillo E., García L., Abad J.L. (2009). Requirement of IFN-gamma-mediated indoleamine 2,3-dioxygenase expression in the modulation of lymphocyte proliferation by human adipose-derived stem cells. Tissue Eng. Part. A.

[40] Torres Crigna A., Uhlig S., Elvers-Hornung S., Klüter H., Bieback K. (2020). Human Adipose Tissue-Derived Stromal Cells Suppress Human, but Not Murine Lymphocyte Proliferation, via Indoleamine 2,3-Dioxygenase Activity. Cells.

[41] Chen G., Goeddel D.V. (2002). TNF-R1 signaling: A beautiful pathway. Science.

[42] Vodyanik M.A., Yu J., Zhang X., Tian S., Stewart R., Thomson J.A., Slukvin I.I. (2010). A mesoderm-derived precursor for mesenchymal stem and endothelial cells. Cell Stem Cell.

[43] Gunne-Braden A., Sullivan A., Gharibi B., Sheriff R.S.M., Maity A., Wang Y.F., Edwards A., Jiang M., Howell M., Goldstone R. (2020). GATA3 Mediates a Fast, Irreversible Commitment to BMP4-Driven Differentiation in Human Embryonic Stem Cells. Cell Stem Cell.

[44] Bottasso-Arias N., Leesman L., Burra K., Snowball J., Shah R., Mohanakrishnan M., Xu Y., Sinner D. (2022). BMP4 and Wnt signaling interact to promote mouse tracheal mesenchyme morphogenesis. Am. J. Physiol. Lung Cell Mol. Physiol..

[45] Soshnikova N., Zechner D., Huelsken J., Mishina Y., Behringer R.R., Taketo M.M., Crenshaw E.B., Birchmeier W. (2003). Genetic interaction between Wnt/beta-catenin and BMP receptor signaling during formation of the AER and the dorsal-ventral axis in the limb. Genes Dev..

[46] Itasaki N., Hoppler S. (2010). Crosstalk between Wnt and bone morphogenic protein signaling: A turbulent relationship. Dev. Dyn..

[47] Eto S., Goto M., Soga M., Kaneko Y., Uehara Y., Mizuta H., Era T. (2018). Mesenchymal stem cells derived from human iPS cells via mesoderm and neuroepithelium have different features and therapeutic potentials. PLoS ONE.

[48] Zhou Y., Yamamoto Y., Xiao Z., Ochiya T. (2019). The Immunomodulatory Functions of Mesenchymal Stromal/Stem Cells Mediated via Paracrine Activity. J. Clin. Med..

[49] Harrell C.R., Fellabaum C., Jovicic N., Djonov V., Arsenijevic N., Volarevic V. (2019). Molecular Mechanisms Responsible for Therapeutic Potential of Mesenchymal Stem Cell-Derived Secretome. Cells.

[50] Rubtsov Y., Goryunov K., Romanov A., Suzdaltseva Y., Sharonov G., Tkachuk V. (2017). Molecular Mechanisms of Immunomodulation Properties of Mesenchymal Stromal Cells: A New Insight into the Role of ICAM-1. Stem Cells Int..

[51] Suzdaltseva Y.u.G., Goryunov K.V., Rubtsov Y.u.P. (2018). The Role of Intercellular Contacts in Induction of Indolamine-2,3-Dioxygenase Synthesis in MMSC from Adipose Tissue. Cell Tissue Biol..

[52] Lau C.I., Yánez D.C., Papaioannou E., Ross S., Crompton T. (2022). Sonic Hedgehog signalling in the regulation of barrier tissue homeostasis and inflammation. FEBS J..

[53] Le H., Kleinerman R., Lerman O.Z., Brown D., Galiano R., Gurtner G.C., Warren S.M., Levine J.P., Saadeh P.B. (2008). Hedgehog signaling is essential for normal wound healing. Wound Repair. Regen..

[54] Suh H.N., Han H.J. (2015). Sonic hedgehog increases the skin wound-healing ability of mouse embryonic stem cells through the microRNA 200 family. Br. J. Pharmacol..

[55] Wang X., Kimbrel E.A., Ijichi K., Paul D., Lazorchak A.S., Chu J., Kouris N.A., Yavanian G.J., Lu S.J., Pachter J.S. (2014). Human ESC-derived MSCs outperform bone marrow MSCs in the treatment of an EAE model of multiple sclerosis. Stem Cell Rep..

[56] Hawkins K.E., Corcelli M., Dowding K., Ranzoni A.M., Vlahova F., Hau K.L., Hunjan A., Peebles D., Gressens P., Hagberg H. (2018). Embryonic Stem Cell-Derived Mesenchymal Stem Cells (MSCs) Have a Superior Neuroprotective Capacity Over Fetal MSCs in the Hypoxic-Ischemic Mouse Brain. Stem Cells Transl. Med..

[57] Lian Q., Zhang Y., Zhang J., Zhang H.K., Wu X., Zhang Y., Lam F.F., Kang S., Xia J.C., Lai W.H. (2010). Functional mesenchymal stem cells derived from human induced pluripotent stem cells attenuate limb ischemia in mice. Circulation.

[58] Rinkevich Y., Walmsley G.G., Hu M.S., Maan Z.N., Newman A.M., Drukker M., Januszyk M., Krampitz G.W., Gurtner G.C., Lorenz H.P. (2015). Skin fibrosis. Identification and isolation of a dermal lineage with intrinsic fibrogenic potential. Science.

[59] Jiang D., Correa-Gallegos D., Christ S., Stefanska A., Liu J., Ramesh P., Rajendran V., De Santis M.M., Wagner D.E., Rinkevich Y. (2018). Two succeeding fibroblastic lineages drive dermal development and the transition from regeneration to scarring. Nat. Cell Biol..

[60] Mascharak S., des Jardins-Park H.E., Davitt M.F., Griffin M., Borrelli M.R., Moore A.L., Chen K., Duoto B., Chinta M., Foster D.S. (2021). Preventing Engrailed-1 activation in fibroblasts yields wound regeneration without scarring. Science.

[61] Mbongue J.C., Nicholas D.A., Torrez T.W., Kim N.S., Firek A.F., Langridge W.H. (2015). The Role of Indoleamine 2, 3-Dioxygenase in Immune Suppression and Autoimmunity. Vaccines.

[62] Sorgdrager F.J.H., Naudé P.J.W., Kema I.P., Nollen E.A., Deyn P.P. (2019). Tryptophan Metabolism in Inflammaging: From Biomarker to Therapeutic Target. Front. Immunol..

[63] Wilgus T.A., Bergdall V.K., Tober K.L., Hill K.J., Mitra S., Flavahan N.A., Oberyszyn T.M. (2004). The impact of cyclooxygenase-2 mediated inflammation on scarless fetal wound healing. Am. J. Pathol..

[64] Zhou B., Lin W., Long Y., Yang Y., Zhang H., Wu K., Chu Q. (2022). Notch signaling pathway: Architecture, disease, and therapeutics. Signal Transduct. Target. Ther..

[65] Wang L., Chen Y.G. (2016). Signaling Control of Differentiation of Embryonic Stem Cells toward Mesendoderm. J. Mol. Biol..

[66] Gumede D.B., Abrahamse H., Houreld N.N. (2024). Targeting Wnt/beta-catenin signaling and its interplay with TGF-beta and Notch signaling pathways for the treatment of chronic wounds. Cell Commun. Signal..

[67] Hu H.H., Cao G., Wu X.Q., Vaziri N.D., Zhao Y.Y. (2020). Wnt signaling pathway in aging-related tissue fibrosis and therapies. Ageing Res. Rev..

[68] Burgy O., Königshoff M. (2018). The WNT signaling pathways in wound healing and fibrosis. Matrix Biol..

