# Supplementary material for: Initial WNT/β-Catenin or BMP Activation Modulates Inflammatory Response of Mesodermal Progenitors Derived from Human Induced Pluripotent Stem Cells
Source: Cells. 2024 Nov 4;13(21):1820. doi: 10.3390/cells13211820 (PMC11545028; doi:10.3390/cells13211820)

Supplementary Materials

Initial WNT/β-catenin or BMP activation modulates inflammatory response of mesodermal progenitors derived from human induced pluripotent stem cells

Yulia Suzdaltseva *, Anastasia Selezneva, Nikita Sergeev and Sergey L. Kiselev

Table S1. Human-specific oligonucleotide primers

Gene		Nucleotide sequence 5'–3'
POU5F1	F	CAAAGCAGAAACCCTCGTGC
	R	TGATCTGCTGCAGTGTGGG
SOX2	F	TTTGTCGGAGACGGAGAAGC
	R	TAACTGTCCATGCGCTGGTT
NANOG	F	ATCTGCTTATTCAGGACAGCCC
	R	AAAGGCTGGGGTAGGTAGGT
BRY	F	CCAATGGGGGTGGCTTCTT
	R	ATGGGTGAGGGGTGTGTAGT
MIXL1	F	CGAGTCCAGGATCCAGCTTTT
	R	CTCCAACCCCGTTTGGTTCG
HAND1	F	ATCAAGACTCTGCGCCTAGC
	R	AGGAAAACCTTCGTGCTGCT
HAND2	F	GCCAAGGACGACCAGAATGG
	R	GGTTTTCTTGTCGTTGCTGCT
FOXF1	F	CGGCTTCCGAAGGAAATGC
	R	CGGCAAGTGGCCGTTCAT
BMP4	F	GGAGCTTCCACCACGAAGAA
	R	GGAAGCCCCTTTCCCAATCA
WNT5A	F	TAGCCTGAAGACATGCTGGC
	R	TGACCTGTACCAACTTGCCC
MEOX1	F	TTGCGGTAAACCTGGACCTC
	R	CTTGGAGAGGCTGTGGAGTC
DLL1	F	CACCGCTATGTGTGCGAGTG
	R	GTTCATGGTCTCCGTCTCCC
MSGN1	F	TCCCCCTCTCAGAGCCTTTC
	R	TGACTTTGGTGCCCTTCTGG
PDGFRB	F	TGGCCCTCAAAGGCGAG
	R	TTCTTTGCGGGGGTATGTCC
KDR	F	GTAACCCGGAGTGACCAAGG

	R	AACCAAGGTACTTCGCAGGG
APLNR	F	ACCTACACGTACCGGGACTA
	R	GTGGTGCGTAACACCATGAC
TBX6	F	TCCATCGTGTCAAGCTCACC
	R	GTGGGTTCTGGTAGGCTGTC
GLI1	F	CTCCTCCCGAAGGACAGGTA
	R	AGAGGGAGGTGGGGTATGTC
EN1	F	CTGGGTGTACTGCACACGTT
	R	CTGGAACTCCGCCTTGAGTC
CD274	F	CTGGCATTTGCTGAACGCATTT
	R	TTGGAGGATGTGCCAGAGGT
TNFAIP6	F	AGATGACCCAGGTTGCTTGG
	R	TTGGAAACCTCCAGCTGTCA
TGFB1	F	TGATGTCACCGGAGTTGTGC
	R	TGAACCCGTTGATGTCCACTT
TGFB3	F	TCATGATGATTCCCCCACACC
	R	CTCCAAGTTGCGGAAGCAGT
CTNNB1	F	ATGACTCGAGCTCAGAGGGT
	R	ATTGCACGTGTGGCAAGTTC
IFNGR2	F	CAGCAGCTACTCATGACCCTC
	R	TGTCGGCCGTGAACCATTTA
TNFRSF1A	F	ATTGGACTGGTCCCTCACCT
	R	CACTCCCTGCAGTCCGTATC
IDO1	F	GGAGGACATGCTGCTCAGTT
	R	AGCTTTCACACAGGCGTCAT
NOS2	F	CAATGTGGAGAAAGCCCCCT
	R	CCTGGGTCCTCTGGTCAAAC
PTGS2	F	TTGCATTCTTTGCCCAGCAC
	R	ACCGTAGATGCTCAGGGACT
ICAM1	F	AGGTTGAACCCCACAGTCAC
	R	CTGAGACCTCTGGCTTCGTC
GAPDH	F	ATGGCAAATTCCATGGCACC
	R	GACTCCACGACGTACTCAGC

Table S2. Antibodies and their final dilutions used for immunofluorescence analysis and flow cytometry.

Antibody	Dilution	Catalog number, vendor
Anti – human β-catenin	1:200	PAC519Hu01, Cloud-Clone corp., Beijing, China
Anti – human HAND1	1:200	PAB021Hu01, Cloud-Clone corp., Beijing, China
Anti-human DLL3	1:100	SAA0168, Antibody System, Schiltigheim, France
Anti –-Human CD90	1:200	561972, BD Bioscience, San Jose, CA, USA
Anti –-Human CD73	1:200	561260, BD Bioscience, San Jose, CA, USA
Anti –-Human CD105	1:200	568752, BD Bioscience, San Jose, CA, USA
Anti –-Human CD14	1:200	562690, BD Bioscience, San Jose, CA, USA
Anti –-Human CD34	1:200	568771, BD Bioscience, San Jose, CA, USA

Anti –-Human CD45	1:200	340943, BD Bioscience, San Jose, CA, USA
Alexa Fluor® 647 Mouse IgG1, κ Isotype Control	1:200	566011, BD Bioscience, San Jose, CA, USA
APC Mouse IgG1, Isotype κ Isotype Control	1:200	340442, BD Bioscience, San Jose, CA, USA
PerCP-Cy™5.5 Mouse IgG1, κ Isotype Control	1:200	347202, BD Bioscience, San Jose, CA, USA
PE-Cy™7 Mouse IgG1, κ Isotype Control	1:200	348788, BD Bioscience, San Jose, CA, USA
Goat anti-Rabbit IgG, Alexa Fluor 594	1:500	ab150084, Abcam, Cambridge, UK
Goat anti-Rabbit IgG, Alexa Fluor 488	1:500	ab150077, Abcam, Cambridge, UK

Figure S1. Cross-activation of HAND1 expression in iPSCs treated with CHIR and BMP4. (a) Expression dynamics of HAND1 during mesodermal differentiation of iPSCs. (b) Flow cytometry analysis shows comparable HAND1+ and HAND1− subpopulations in iPSCs treated with BMP4 and CHIR on day 2 of differentiation and significant differences between them on day 6. $\chi 2$, $**p < 0.01$.

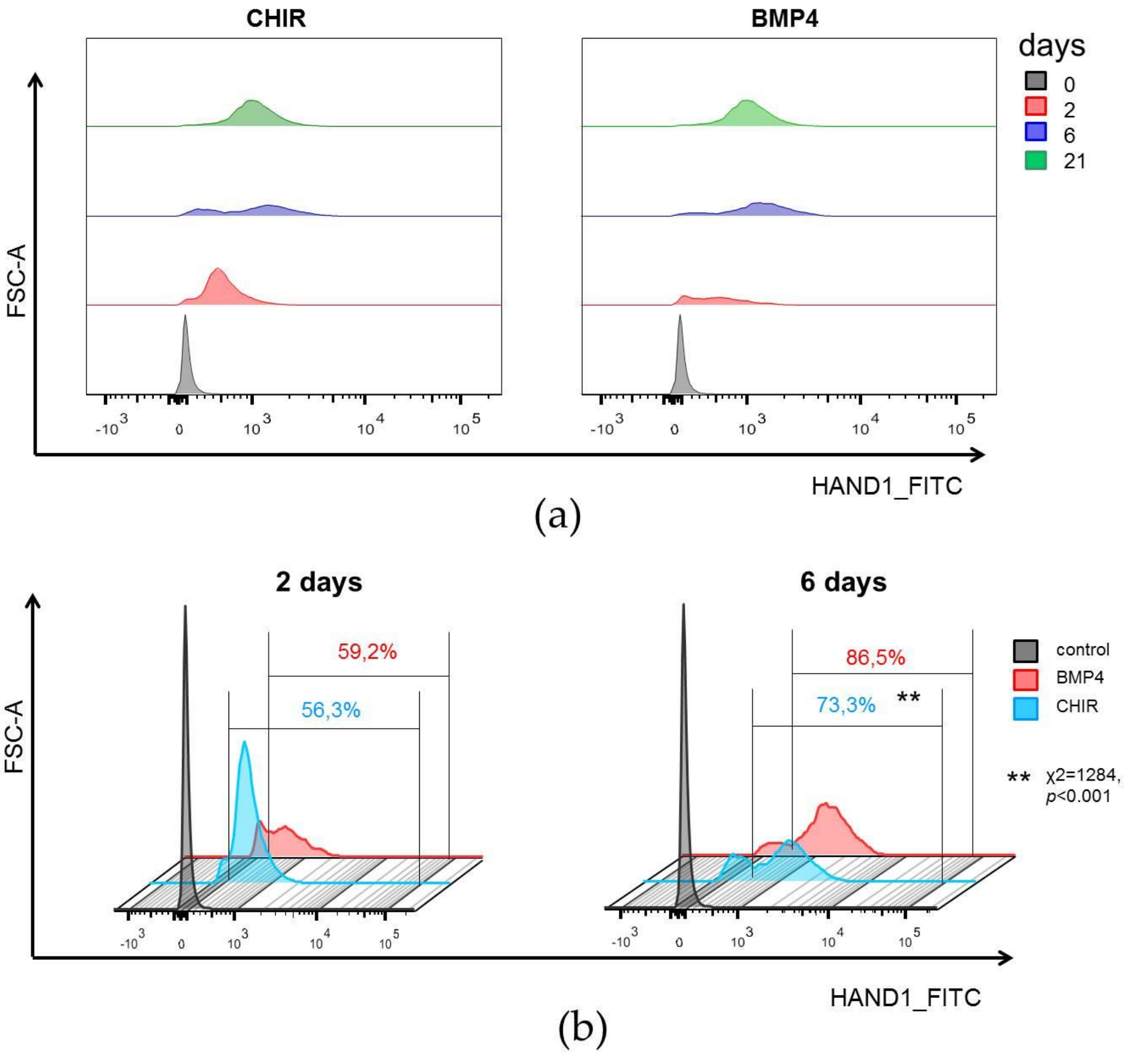

Supplement: Supplementary file 1 [file cells-13-01820-s001.zip › cells-3268864-supplementary.pdf]
